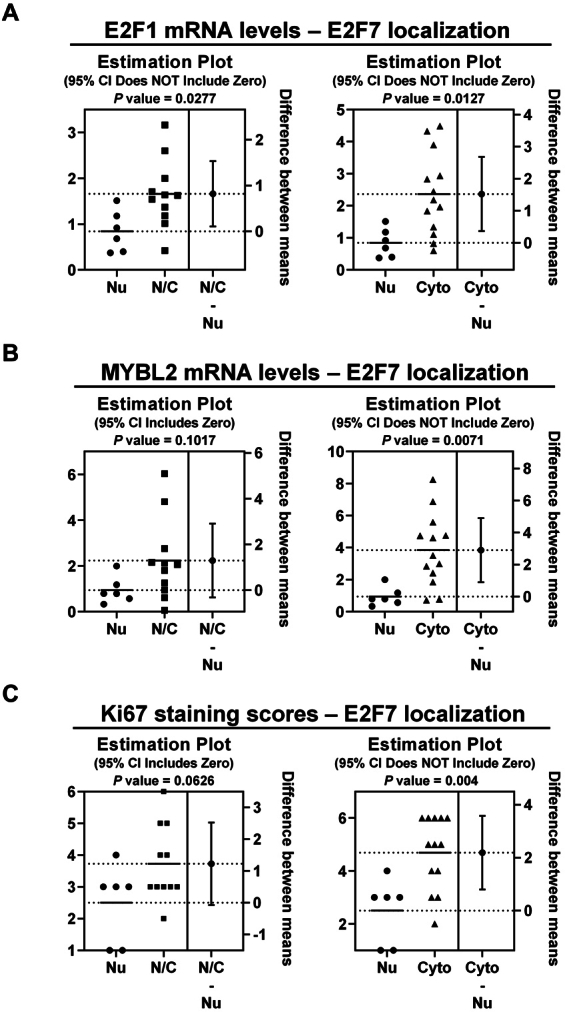# E2F1 and E2F7 regulate gastric cancer cell proliferation, respectively, through transcriptional activation and transcriptional repression of MYBL2

**DOI:** 10.1016/j.jbc.2024.108027

**Published:** 2024-11-27

**Authors:** Tianyi Wu, Fengli Jiang, Fan Wu, Guoliang Zheng, Yang Li, Lizhao Wu

**Affiliations:** 1 Department of Pathophysiology, College of Basic Medical Sciences, China Medical University, Shenyang, China; 2 Department of Gynecology, Shengjing Hospital of China Medical University, Shenyang, China; 3 Department of Gastric Surgery, Cancer Hospital of China Medical University (Liaoning Cancer Hospital and Institute), Shenyang, China

**Keywords:** gastric cancer, transcription regulation, cell proliferation, E2F transcription factor, MYBL2, nucleocytoplasmic distribution, tumor cell biology

## Abstract

Gastric cancer (GC) is the most common malignant tumor of the digestive tract. However, the molecular pathogenesis is not well understood. Through bioinformatic analysis and analyzing clinical tissue samples, we found that E2F1 and E2F7 as well as their potential downstream target MYBL2 were all upregulated in GC tissues and that their expressions correlated with patient prognosis. While knockdown of E2F1 or MYBL2 inhibited cell proliferation and promoted apoptosis, knockdown of E2F7 promoted cell proliferation but had no effects on apoptosis. Chromatin immunoprecipitation and dual luciferase reporter assays demonstrated that MYBL2 was transcriptionally activated and repressed by E2F1 and E2F7, respectively. Importantly, *in vitro* and *ex vivo* experiments demonstrated that the effects of E2F1 and E2F7 on GC cell proliferation were significantly attenuated by reversely modulating MYBL2 expression, indicating that MYBL2 is a direct and functionally relevant target of E2F1 and E2F7 in GC cells. Furthermore, the effects of E2F1 and E2F7 on GC cell proliferation through transcriptional regulation of MYBL2 can be mediated by the PI3K/AKT signaling pathway. Interestingly, we found differential nucleocytoplasmic distribution of E2F7 in GC cells with functional relevance. Taken together, our data suggest that targeted therapies of GC may be achieved from three different angles, E2F1, E2F7, and MYBL2 themselves, E2F1/E2F7 expression balance, and E2F7 nuclear localization.

Gastric cancer (GC) is the fifth most common cancer and the fifth leading cause of cancer death worldwide (1). Although the incidence of GC has declined in Western countries in recent years, it remains a serious public health problem in developing countries (1, 2). Therefore, identifying critical molecules/pathways responsible for the pathogenesis of GC is beneficial for its prevention, diagnosis, and treatment.

The adenoviral early region two binding factor (E2F) family is encoded by eight different genes (E2F1–E2F8) (3) and plays important roles in controlling various biological processes involved in cancer development (3, 4). While E2F1, E2F2, and E2F3 are transcriptional activators, E2F4, E2F5, and E2F6 are typical transcriptional repressors, and E2F7 and E2F8 are atypical transcriptional repressors (3). E2F1–E2F6 form heterodimers with dimerization partner proteins and bind to DNA in a sequence-specific manner (5). In the late G1 and S phases, E2F1–E2F3 form complexes with retinoblastoma (RB), preventing E2F-mediated gene activation (6). E2F6–E2F8 do not bind to pocket proteins or dimerization partners but form homodimers or heterodimers through their two DNA-binding domains, inhibiting cell proliferation and hindering target gene expression (7, 8, 9).

Among the E2F family members, E2F1 is currently the most widely studied. Several earlier studies using transgenic mouse models or *in vitro* systems suggested that the role of E2F1 in tumorigenesis was bidirectional, as E2F1 either promoted or inhibited tumorigenesis depending on the dominant signaling pathways and the cell types involved (10, 11, 12, 13, 14). In GC, E2F1 is highly upregulated and positively correlated with tumor malignancies (15). Several research groups have attempted to define the effects of E2F1 overexpression or knockdown on the tumorigenicity of GC cells but yielded inconsistent results (16, 17, 18, 19, 20, 21). Thus, the precise role and mechanisms of E2F1 in GC remain unresolved.

Previous studies have shown that atypical E2F repressors and E2F1 could be mutually regulated. For example, E2F7 is induced by E2F1 in the late G1 phase of the cell cycle, peaks in the S phase, and decreases in the G2 phase (22). Moreover, E2F7 and E2F8 can bind to the E2F1 promoter and repress its transcription in cell culture systems (7, 8, 9, 23, 24, 25). Importantly, atypical E2F repressor-mediated repression of E2F1 has *in vivo* biological relevance, as loss of E2F7 and E2F8 in mice synergizes to derepress E2F1, leading to increased apoptosis and embryonic lethality (26). In addition, in two human cancer cell lines (U2O and HeLa), E2F7 recruited the corepressor C-terminal binding protein to repress the transcription of E2F1 in a C-terminal binding protein-dependent manner, leading to reduced cell proliferation (27, 28). Although E2F7 has been reported to act as a tumor suppressor in murine skin cancer and liver cancer (29, 30), other data are consistent with its oncogenic role in murine non-small cell lung cancer (31) and several other human cancers (32, 33, 34, 35, 36). Bioinformatic analysis revealed high expression of E2F7 in GC and its positive correlation with good prognosis (37, 38). However, the specific role of E2F7 in GC has not been defined.

In murine embryonic fibroblasts, both MYB proto-oncogene like 2 (MYBL2) and E2F1 promote cell cycle progression, and their expression levels are positively correlated (39, 40). In quiescent, immortalized but non-transformed murine and human cells, MYBL2 promoter activity appears to be repressed by E2F (41). Interestingly, MYBL2 has also been implicated in various types of human cancers. The expression levels of MYBL2 were positively correlated with those of E2F1 in hepatocellular carcinomas, and downregulation of E2F1 in hepatocellular carcinoma cells reduced MYBL2 expression (42). In addition, in A431 human epidermoid carcinoma cells and Chinese hamster ovary cells, E2F1 bound to the MYBL2 promoter and activated its transcription (43). Furthermore, a recent study using only clinical samples showed that higher levels of MYBL2 were associated with poor prognosis, poor differentiation, and lymph node metastasis (44), suggesting that MYBL2 may have an oncogenic function in GC. However, neither the precise role of MYBL2 nor its potential association with E2Fs in GC has been established.

In the present study, we aimed to define the role and explore the underlying mechanisms of E2F1, E2F7, and MYBL2 in GC and to establish the relationships among the two E2F transcription factors and their potential downstream target MYBL2 in GC cells.

## Results

### E2F1, E2F7, and MYBL2 are highly expressed in GC tissues

**Figure 1:**
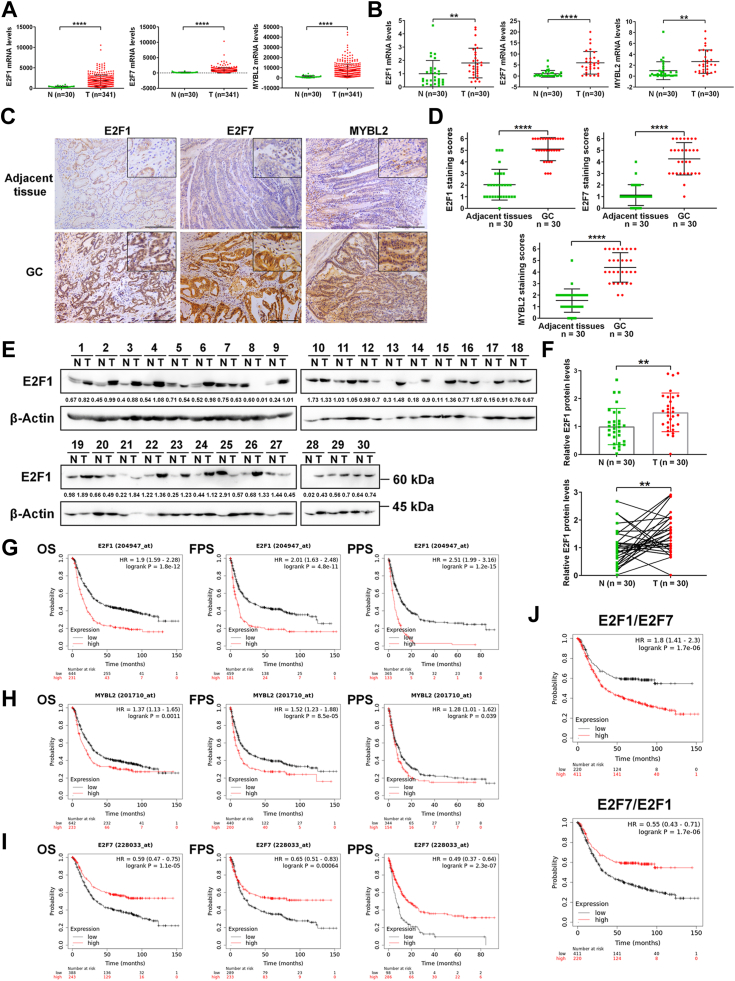
**E2F1, E2F7 and MYBL2 are highly expressed in GC tissues and correlated with the prognosis of GC patients.***A*, mRNA levels of E2F1, E2F7 and MYBL2 in GC tissues and normal stomach tissues in TCGA dataset. *B*, mRNA levels of E2F1, E2F7, and MYBL2 evaluated by RT-qPCR (n = 3) in 30 GC tissues and their paracancerous tissues. *C*, representative pictures of IHC for E2F1, E2F7, and MYBL2 in clinical GC samples. Scale bars: 100 μm. *D*, scatter plots of E2F1, E2F7, and MYBL2 IHC staining scores on 30 paired clinical samples (N = 30, T = 30). *E*, E2F1 WBs for 30 paired clinical samples (N = 30, T = 30). In this paper, all numbers beneath each band (*i.e.* 0.67) represent the relative intensities of the WB bands, normalized to the intensities of their corresponding internal reference bands. *F*, quantification of E2F1 protein levels in (*E*). *Top*: unpaired comparisons; *Bottom*: paired comparisons. *G*–*I*, Kaplan Meier curves for OS (*left* column), FPS (*middle* column) and PPS (*right* column) for patients with GC having high or low mRNA levels of E2F1 (*G*), MYBL2 (*H*), and E2F7 (*I*). *J*, Kaplan-Meier OS curves for GC patients with high or low ratios of E2F1/E2F7 mRNA levels (*top* panel) or E2F7/E2F1 mRNA levels (*bottom* panel) corresponding to OS in GC patients. In this paper, ∗*p* < 0.05, ∗∗*p* < 0.01, ∗∗∗*p* < 0.001, ∗∗∗∗*p* < 0.0001, ns: not significant.

To understand the roles of E2F1, E2F7, and MYBL2 in GC, we first evaluated their differential gene expression in GC tissues compared with normal tissues from the TCGA dataset. As shown in Fig. 1*A*, the mRNA levels of E2F1, E2F7 and MYBL2 in GC tissues were all higher than those in normal tissues. To validate the high expression status of these three genes in GC tissues from a public dataset, we used reverse-transcription quantitative PCR (RT‒qPCR) to evaluate differential gene expression in 30 clinical paracancerous samples and 30 clinical GC samples. Consistent with those in the public dataset, the mRNA levels of E2F1, E2F7, and MYBL2 in our clinical GC tissues were also significantly higher than those in paracancerous tissues (Fig. 1*B*).

Next, we performed immunohistochemical (IHC) staining to evaluate the protein levels of E2F1, E2F7, and MYBL2 in 30 paired clinical samples. The results showed that there was generally greater staining for E2F1, E2F7, and MYBL2 in GC tissues than in paracancerous tissues (Fig. 1, *C* and *D*; Tables S1–S3). In addition, we used Western blot (WB) to evaluate E2F1 protein levels in 30 paired clinical samples and confirmed that the E2F1 protein levels in the GC tissues were generally greater than those in the paracancerous tissues (Fig. 1, *E* and *F*, top). Among the 30 pairs, 21 had relatively high E2F1 protein levels in GC tissues (Fig. 1*F*, bottom). Thus, both mRNA levels and protein levels of E2F1, E2F7, and MYBL2 are higher in GC tissues than those in paracancerous tissues.

### Expression levels of E2F1, E2F7 and MYBL2 in GC are related to the prognosis

To evaluate the clinical relevance of E2F1, E2F7, and MYBL2 in GC, we used the Kaplan–Meier Plotter online tool to establish correlations between their mRNA levels and the prognosis of patients with GC. Survival curves showed that higher levels of E2F1 or MYBL2 mRNA were correlated with poor prognosis in patients with GC, as evidenced by shorter overall survival (OS), first-progression survival (FPS), and post-progression survival (PPS) (Fig. 1, *G* and *H*). Conversely, higher levels of E2F7 mRNA were correlated with good prognosis (*i.e.*, longer OS, FPS, and PPS) (Fig. 1*I*). Because E2F1 and E2F7 appeared to have opposite effects on prognosis (Fig. 1, *G* and *I*) and MYBL2 is a potential E2F target gene in GC cells, we next established correlations between the ratio of E2F1 and E2F7 mRNA levels and the prognosis of patients with GC. As shown in Fig. 1*J*, higher E2F1/E2F7 mRNA ratios were correlated with poor prognosis, whereas higher E2F7/E2F1 mRNA ratios were correlated with good prognosis, further supporting the oncogenic role of E2F1 and the tumor suppressor role of E2F7 in GC.

### Knockdown of E2F1 or MYBL2 inhibits GC cell proliferation, whereas knockdown of E2F7 promotes cell proliferation

**Figure 2:**
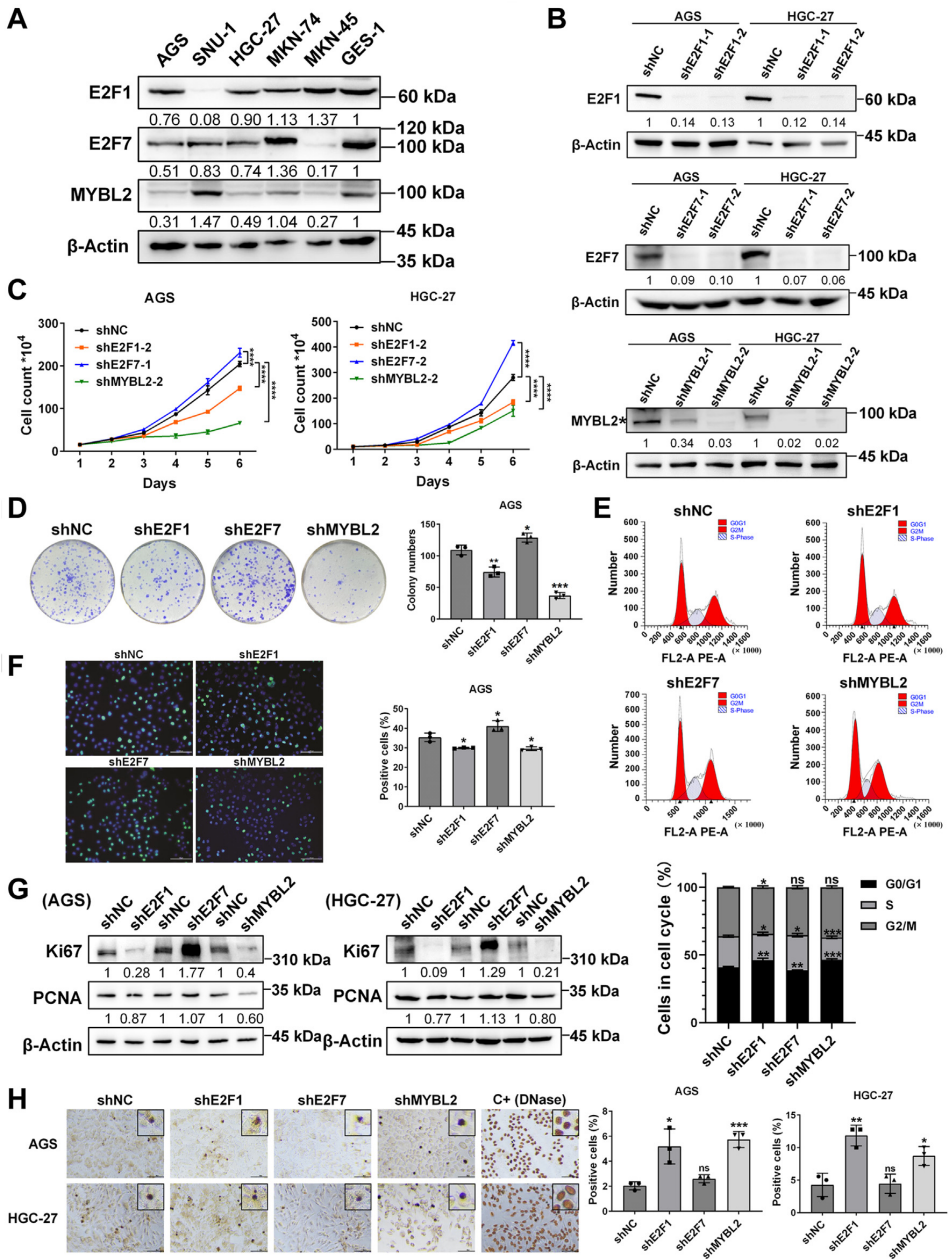
**Effects of knockdown of E2F1, E2F7, or MYBL2 on GC cell proliferation and apoptosis.***A*, protein levels of E2F1, E2F7, and MYBL2 in 5 GC cell lines and one normal gastric mucosal cell line were evaluated by WB (n = 3). In this article, all cellular WB experiments were conducted with three independent biological replicates and the numerical values beneath each band were from the single blot shown. *B*, changes in protein levels after E2F1, E2F7 or MYBL2 knockdown in AGS and HGC-27 cells were evaluated by WB (n = 3). *C*, changes in cell growth curves drawn by cell counting after E2F1, E2F7, or MYBL2 knockdown in AGS and HGC-27 cells (n = 4). *D*, effects of E2F1, E2F7, or MYBL2 knockdown on the colony formation ability of AGS cells were assessed by a colony formation assay (n = 3). *E*, changes in cell cycle distributions after E2F1, E2F7, or MYBL2 knockdown in AGS cells were evaluated by flow cytometry (n = 3). *F*, changes in cellular proliferation of AGS cells after knockdown of E2F1, E2F7, or MYBL2 were assessed by EdU immunofluorescence staining (n = 3). Scale bar: 100 μm. *G*, changes in Ki67 and PCNA protein levels after E2F1, E2F7 or MYBL2 knockdown in GC cells were evaluated by WB (n = 3). *H*, changes in levels of apoptosis of AGS and HGC-27 cells after E2F1, E2F7, or MYBL2 knockdown were assessed by a TUNEL assay (n = 3). Positive control group (C+) cells were treated with DNase. Scale bar: 100 μm.

**Supplementary Figure S1:**
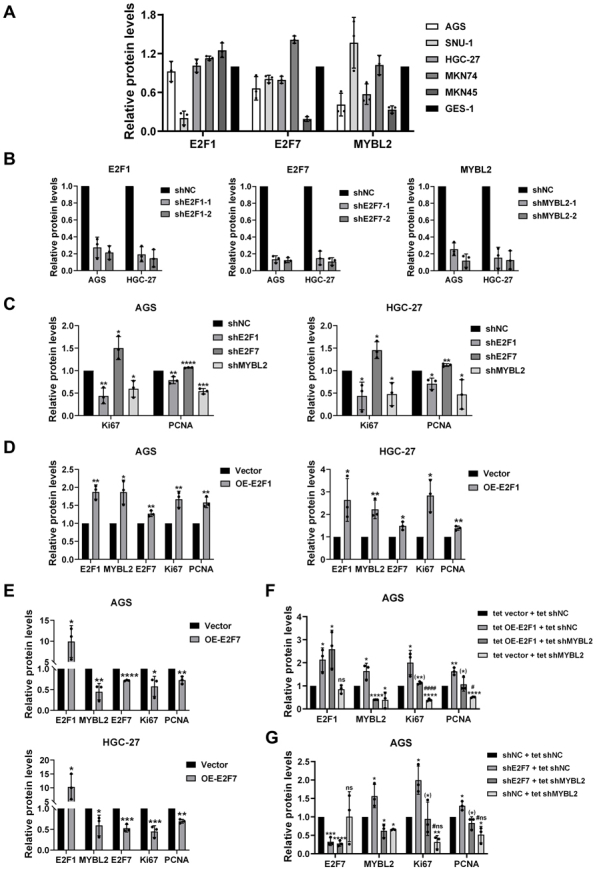


Because bioinformatic analysis and empirical studies of clinical samples support important roles and clinical relevance of E2F1, E2F7, and MYBL2 in GC, we explored the biological effects of genetic intervention involving these three genes in GC cell lines. We first used WB to assess the baseline protein levels in various GC cell lines (*i.e.*, AGS, SNU-1, HGC-27, MKN-74, and MKN-45) and the normal gastric mucosal cell line GES-1 (Fig. 2*A*). Among the 5 GC cell lines, AGS and HGC-27 had relatively moderate levels of the E2F1, E2F7, and MYBL2 proteins (Figs. 2*A*, S1*A*). Therefore, we chose one or both cell lines for subsequent experiments.

**Supplementary Figure S2:**
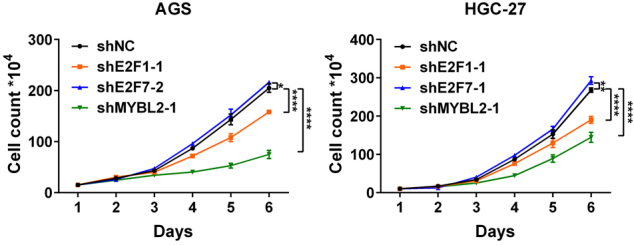


We used lentiviral transfection technology to stably knock down E2F1, E2F7, and MYBL2 in AGS and HGC-27 cells with two shRNA-targeting sequences for each gene. WB showed that E2F1, E2F7, and MYBL2 were significantly downregulated in both GC cell lines (Figs. 2*B*, S1*B*). To assess the effects of E2F1, E2F7, and MYBL2 gene knockdown on GC cell growth, we plotted growth curves *via* cell counting to determine the changes in cell numbers after gene knockdown (Fig. 2*C*). For each duplicate shRNA targeting sequence, we selected those with higher knockdown efficiencies and more significant effects on cell growth to conduct all subsequent experiments. Growth curves corresponding to groups with slightly poor knockdown efficiency and relatively low significant differences in cell growth rates are shown in Fig. S2. As shown in Figures 2*C* and S2, in both cell lines, compared with that in the controls (shNC), levels of cell growth in the E2F1- or MYBL2-knockdown groups were significantly reduced, whereas cell growth in the E2F7-knockdown group was significantly accelerated. Next, we assessed the effects of E2F1, E2F7, and MYBL2 knockdown on the long-term growth potential of AGS cells in a colony formation assay. Compared with the control group, the E2F1- or MYBL2-knockdown group presented fewer colonies, whereas the E2F7-knockdown group presented more colonies (Fig. 2*D*).

Because both cell proliferation and cell death may contribute to cell growth, we assessed the effects of E2F1, E2F7, and MYBL2 on cell proliferation. First, we assessed the cell cycle distribution of AGS cells by flow cytometry. As shown in Figure 2*E*, E2F1 or MYBL2 knockdown led to significant increases in G0/G1 phase cells and significant decreases in S phase cells. Conversely, E2F7 knockdown led to significant decreases in G0/G1 phase cells and significant increases in S phase cells. Additionally, E2F1 knockdown led to a slight but significant decrease in G2/M phase cells. These data suggest that in GC cells, E2F1 and MYBL2 promote the G1‒S transition, whereas E2F7 inhibits the G1‒S transition. Consistent with this notion, EdU immunofluorescence staining revealed that the percentages of EdU-positive cells in the E2F1- and MYBL2-knockdown groups were lower than that in the control group, whereas the percentage of EdU-positive cells in the E2F7-knockdown group was greater (Fig. 2*F*). Furthermore, in AGS and HGC-27 cells, WB revealed that compared with those in the control group, protein levels of the proliferation markers Ki67 and PCNA were lower in the E2F1- or MYBL2-knockdown group, whereas the levels of both proteins were higher in the E2F7-knockdown group (Figs. 2*G*, S1*C*). Therefore, we conclude that E2F1 and MYBL2 promote GC cell growth by enhancing cellular proliferation, whereas E2F7 hinders cell growth by inhibiting cellular proliferation.

### Knockdown of E2F1 or MYBL2 promotes GC cell apoptosis

To evaluate whether apoptosis also contributes to the aforementioned changes in growth rates, we performed a TUNEL assay to assess the effects of gene knockdown on apoptosis in AGS and HGC-27 cells. Although the percentages of TUNEL-positive cells were greater in the E2F1- or MYBL2-knockdown groups than in the control groups, they did not change significantly in the E2F7-knockdown groups (Fig. 2*H*). Therefore, we conclude that E2F1 or MYBL2 knockdown leads to reduced cell growth partly by promoting GC cell apoptosis.

### Overexpression of E2F1 promotes GC cell proliferation and apoptosis

**Supplementary Figure S3:**
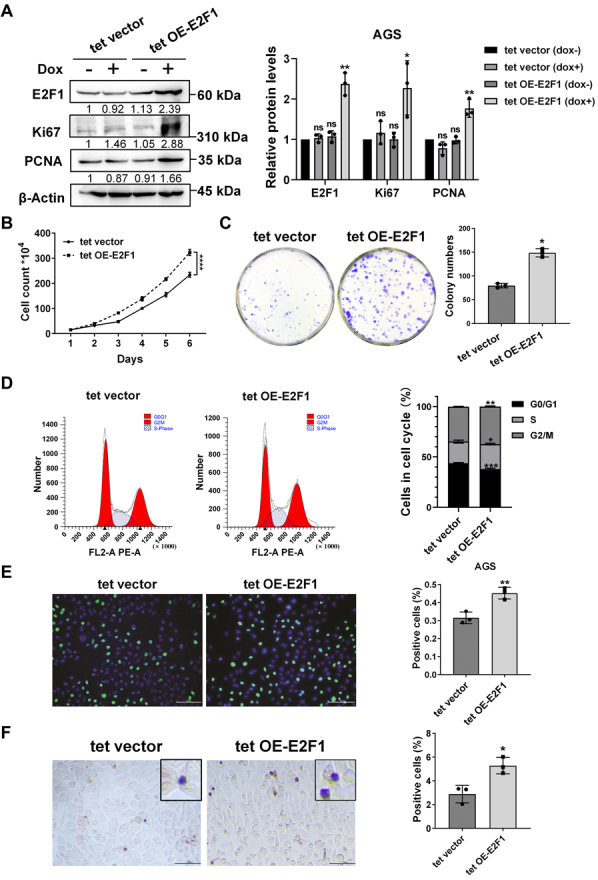


To further assess the effect of E2F1 on GC cells, we stably overexpressed E2F1 in AGS cells using a doxycycline-inducible Tet-On system, as evidenced by WB (Fig. S3*A*). Consistent with the data described above to support a role for E2F1 in promoting GC cell proliferation, stable overexpression of E2F1 in AGS cells led to significant increases in cell growth (Fig. S3*B*), the number of colonies (Fig. S3*C*), the proportions of S and G2/M phase cells (Fig. S3*D*), the percentage of EdU-positive cells (Fig. S3*E*), and the protein levels of Ki67 and PCNA (Fig. S3*A*). Surprisingly, overexpression of E2F1 led to a significant increase in the percentage of TUNEL-positive cells (Fig. S3*F*), suggesting that E2F1 promotes apoptosis in GC cells. Considering that the overexpression of E2F1 resulted in significant increases in cell numbers (Fig. S3*B*), the effect of E2F1 overexpression on promoting cell proliferation is predominant relative to its effect on promoting apoptosis.

### E2F1 upregulates E2F7 and MYBL2, whereas E2F7 downregulates E2F1 and MYBL2 in GC cells

**Figure 3:**
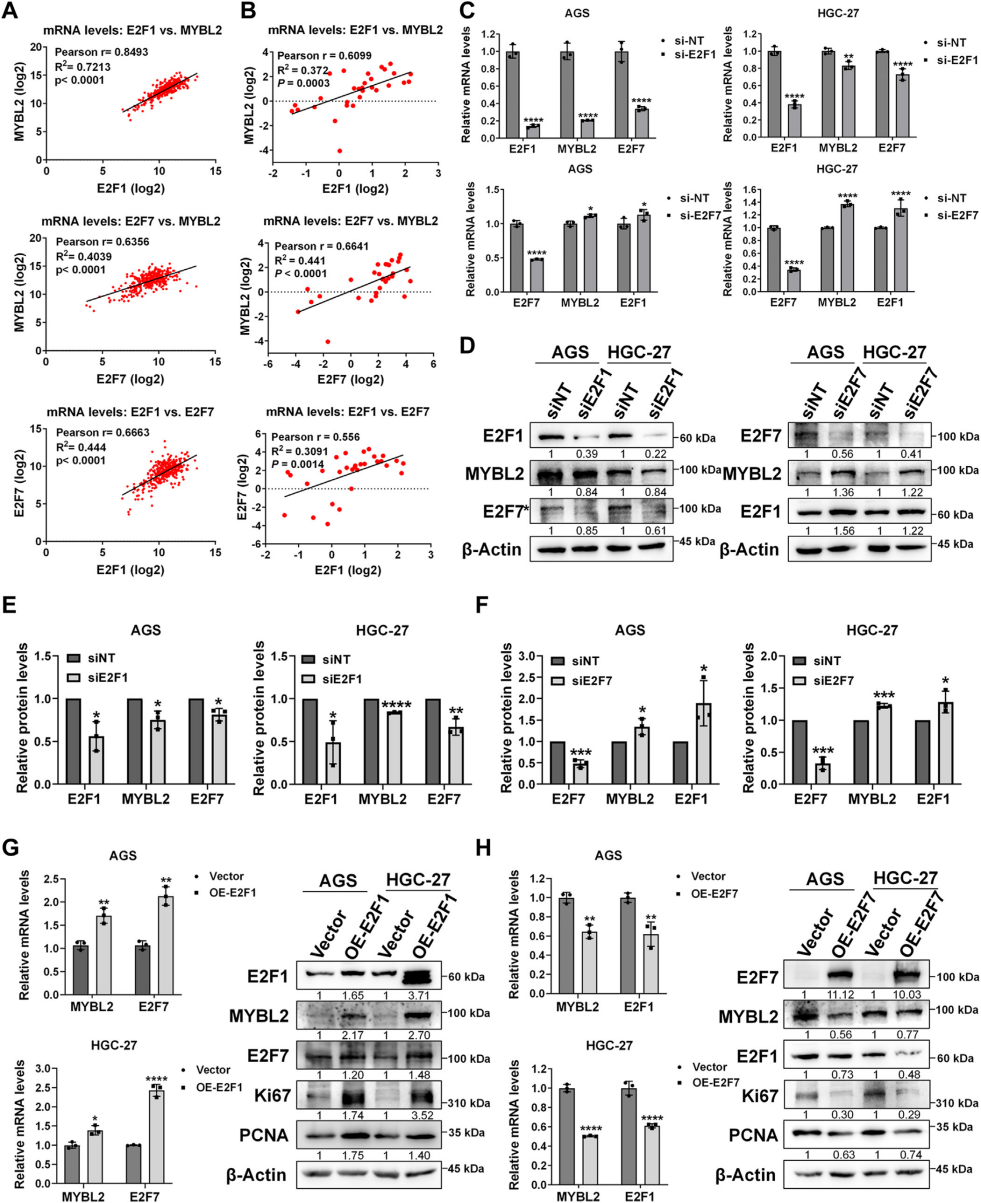
**E2F1 upregulates E2F7 and MYBL2, whereas E2F7 downregulates E2F1 and MYBL2 in GC cells.***A*, correlation between E2F1, E2F7, and MYBL2 mRNA levels in GC tissues from the TCGA dataset (n = 341). *B*, correlation between E2F1, E2F7, and MYBL2 mRNA levels in clinical GC samples (n = 30). *C*, changes in mRNA levels of E2F1, E2F7 and MYBL2 in AGS and HGC-27 cells following siE2F1 or siE2F7 transient transfections were evaluated by RT-qPCR (n = 3). *D*, changes in E2F1, E2F7 and MYBL2 protein levels in AGS and HGC-27 cells after siE2F1 (*left* panel) and siE2F7 (*right* panel) transient transfections were evaluated by WB (n = 3). *E*, quantification of E2F1, E2F7, and MYBL2 protein levels in AGS and HGC-27 cells after siE2F1 (*left* panel in (*D*)). *F*, quantification of E2F7, E2F1 and MYBL2 protein levels in AGS and HGC-27 cells after siE2F7 (*right* panel in (*D*)). *G*, changes in mRNA levels of MYBL2 and E2F7 after E2F1 overexpression in AGS and HGC-27 cells were evaluated by RT-qPCR (n = 3), and changes in protein levels of E2F1, MYBL2, E2F7, Ki67 and PCNA after E2F1 overexpression were evaluated by WB (n = 3). *H*, changes in mRNA levels of MYBL2 and E2F1 after E2F7 overexpression in AGS and HGC-27 cells were evaluated by RT-qPCR (n = 3), and changes in protein levels of E2F7, MYBL2, E2F1, Ki67 and PCNA after E2F7 overexpression were evaluated by WB (n = 3).

To explore the potential regulatory effects of E2F1, E2F7, and MYBL2, we performed correlation analyses on the gene expression data of 341 GC samples in the TCGA dataset. We found that the mRNA levels of E2F1, E2F7, and MYBL2 were mutually positively correlated (Fig. 3*A*). These correlations were also verified by RT‒qPCR in our 30 GC tissues (Fig. 3*B*). To study the regulatory effects of E2F1 and E2F7 on their potential downstream target gene MYBL2 and the interactions among them in GC cells, we used siRNA to transiently knock down E2F1 or E2F7 in GC cells. We found that in both cell lines, mRNA levels of both MYBL2 and E2F7 were reduced upon E2F1 knockdown (Fig. 3*C*). Conversely, mRNA levels of both MYBL2 and E2F1 were increased upon E2F7 knockdown (Fig. 3*C*). Next, we used WB to evaluate changes in the protein levels (Fig. 3*D*). The quantitative data showed that MYBL2 and E2F7 protein levels were reduced upon E2F1 knockdown in AGS and HGC-27 cells (Fig. 3*E*), which is consistent with the mRNA data shown above (Fig. 3*C*). Upon E2F7 knockdown in both cell lines, protein levels of E2F1 and MYBL2 were increased (Fig. 3, *D* and *F*), which was also consistent with the mRNA data (Fig. 3*C*). Additionally, overexpression of E2F1 in AGS and HGC-27 cells led to increases in both mRNA and protein levels of MYBL2 and E2F7 (Figs. 3*G*, S1*D*), whereas overexpression of E2F7 in both cell lines led to decreases in both mRNA and protein levels of MYBL2 and E2F1 (Figs. 3*H*, S1*E*). Furthermore, Ki67 and PCNA protein levels were increased upon E2F1 overexpression but decreased upon E2F7 overexpression (Figs. 3, *G* and *H*, S1, *D* and *E*), further supporting that E2F1 and E2F7 promote and inhibit GC cell proliferation, respectively.

### E2F1 and E2F7 transcriptionally regulate MYBL2 in GC cells

**Figure 4:**
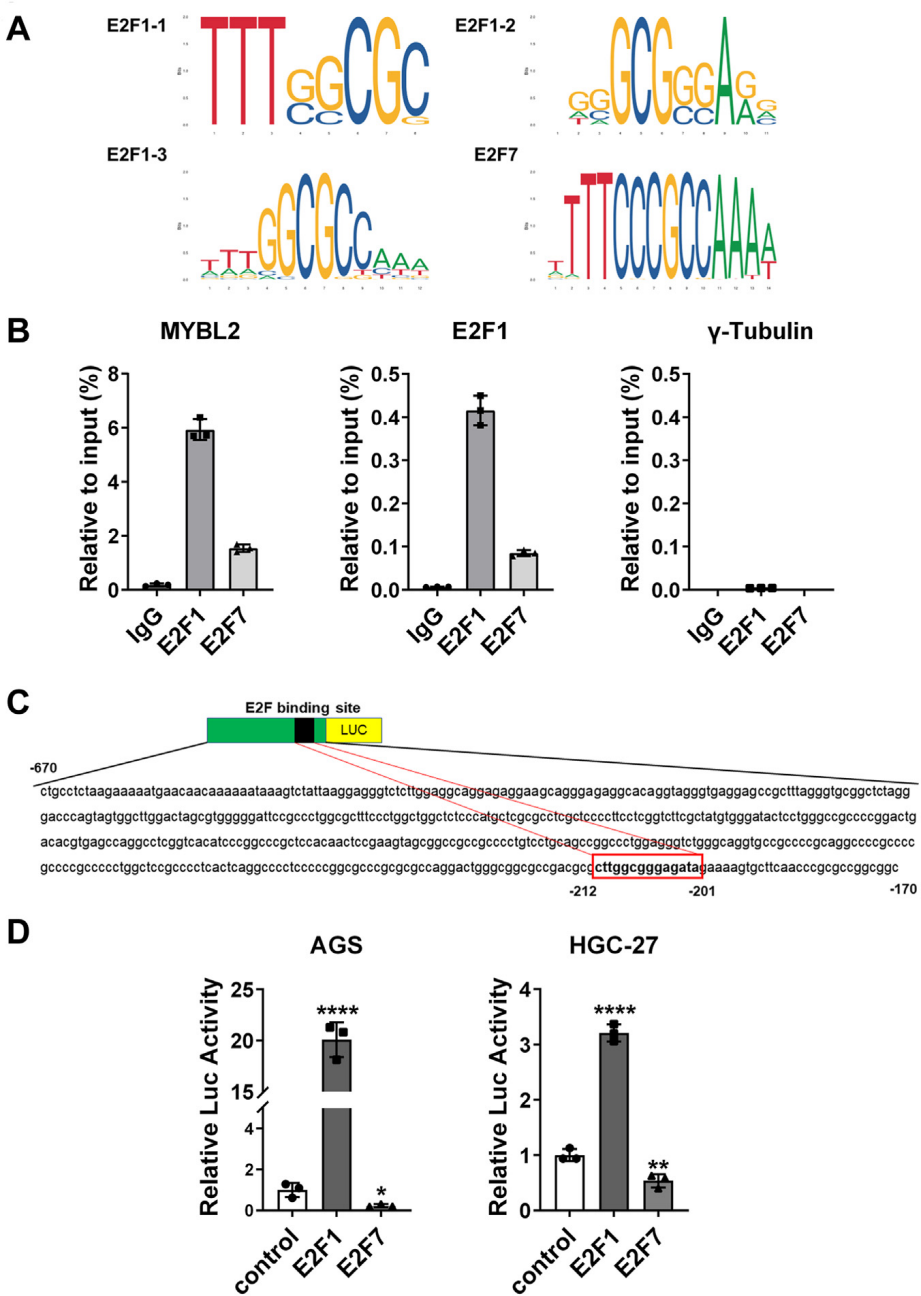
**Both E2F1 and E2F7 bind to the MYBL2 promoter and respectively activate and repress its transcription in GC cells.***A*, consensus binding sites of human E2F1 and E2F7. *B*, E2F1 and E2F7 binding to the MYBL2 and E2F1 promoters in HGC-27 cells was assessed by ChIP-qPCR (n = 3). The γ-tubulin promoter serves as a negative control. *C*, schematic diagram of the MYB-PGL3 plasmid containing the MYBL2 promoter sequence (−670 ∼ −170), with the E2F binding site (−212 ∼ −201) in the red box. *D*, changes of relative luciferase activities following overexpression of E2F1 or E2F7 in AGS and HGC-27 cells were evaluated by a dual-luciferase reporter assay (n = 3).

After showing the mutual correlations among E2F1, E2F7 and MYBL2 in GC cells, we attempted to explore their regulatory mechanisms. A previous study revealed an E2F binding site (CTTGGCGGGAGA) on the human MYBL2 promoter (41), but no studies have demonstrated the binding of E2F1 or E2F7 to the MYBL2 promoter and their ability to regulate the transcriptional activity of MYBL2 in GC cells. The JASPAR website predicted that human E2F1 has three consensus E2F binding sites, whereas human E2F7 has only one (Fig. 4*A*). We used chromatin immunoprecipitation (ChIP) experiments to determine whether E2F1 and/or E2F7 directly bind to the specific E2F binding sites on the MYBL2 and E2F1 promoters in HGC-27 cells. ChIP‒qPCR revealed that both E2F1 and E2F7 bound to the MYBL2 and E2F1 promoters but not to the γ-tubulin promoter in HGC-27 cells (Fig. 4*B*). Next, we determined whether the binding of E2F1 and E2F7 to the MYBL2 promoter in the AGS and HGC-27 cell lines affects the transcriptional regulation of MYBL2. A dual-luciferase reporter assay revealed that, compared with those of the controls, in both cell lines the relative luciferase activities of the E2F1 and E2F7 overexpression groups were significantly increased and decreased, respectively, (Fig. 4, *C* and *D*). Therefore, we conclude that in GC cells, E2F1 and E2F7 transcriptionally activate and repress MYBL2, respectively.

### E2F1 and E2F7 affect GC cell proliferation through the transcriptional regulation of MYBL2 *in vitro* and *ex vivo*

**Figure 5:**
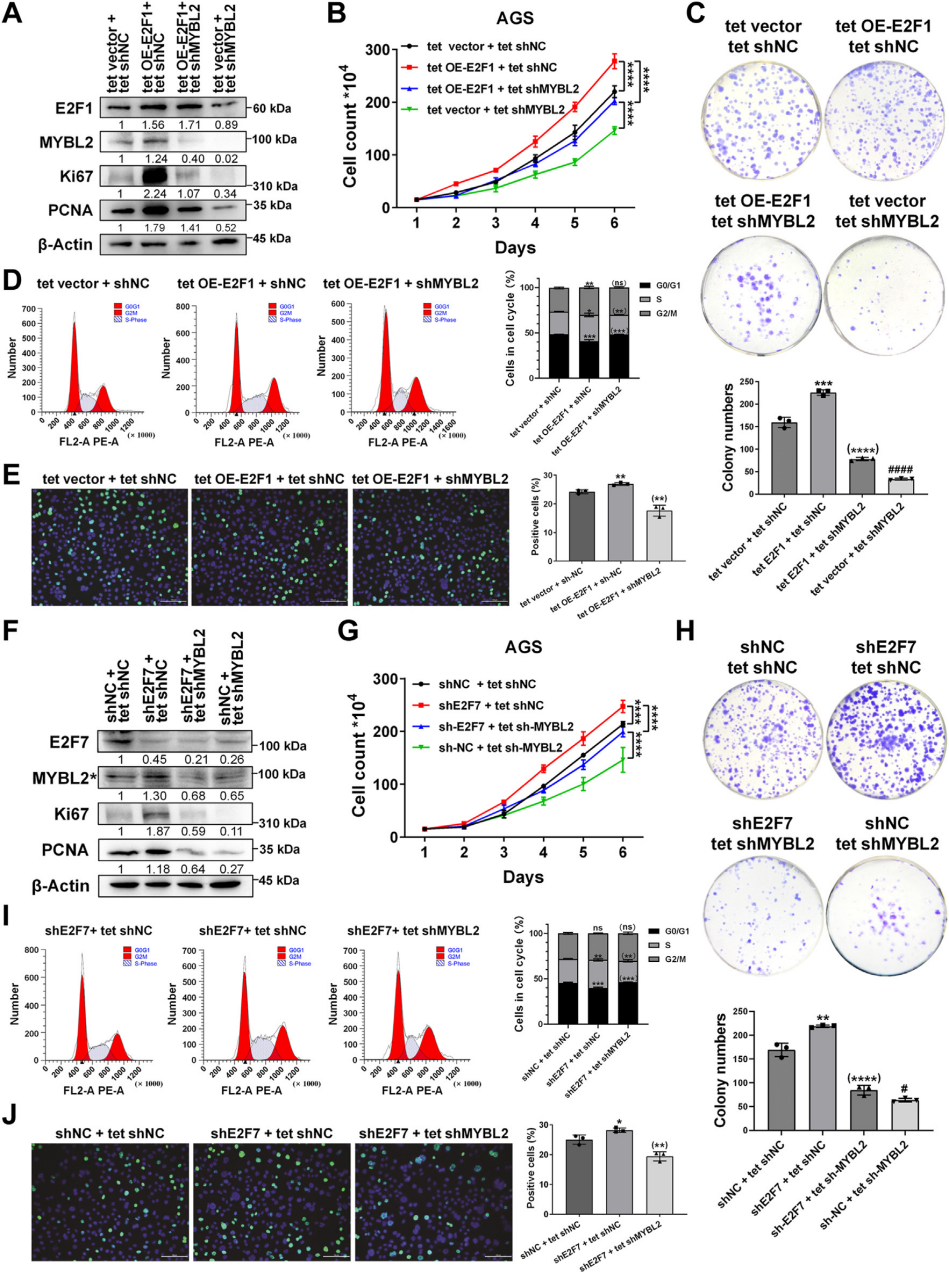
**E2F1 promotes GC cell proliferation by activating MYBL2, whereas E2F7 inhibits GC cell proliferation by repressing MYBL2.***A*, E2F1, MYBL2, Ki67, and PCNA protein levels in tet vector + tet shNC, tet OE-E2F1 + tet shNC, tet OE-E2F1 + shMYBL2, and tet vector + tet shMYBL2 groups were evaluated by WB (n = 3). *B*, cell growth curves were plotted using a cell counting assay (n = 4). *C*, changes in colony forming ability were evaluated by a colony formation assay (n = 3, (∗∗∗∗) *p* < 0.0001 vs. tet OE-E2F1 + tet shNC group, ####*p* < 0.0001 vs. tet OE-E2F1 + tet shMYBL2 group). *D*, cell cycle distributions were assessed by flow cytometry (n = 3, (∗∗) *p* < 0.01/(∗∗∗) *p* < 0.001 vs. tet OE-E2F1 + tet shNC group). *E*, cell proliferation levels were assessed by EdU immunofluorescence assay (n = 3, (∗∗) *p* < 0.01 vs. tet OE-E2F1 + tet shNC group). Scale bar: 100 μm. *F*, E2F7, MYBL2, Ki67 and PCNA protein levels in shNC + tet shNC, shE2F7 + tet shNC, shE2F7 + tet shMYBL2, and shNC + tet shMYBL2 groups were evaluated by WB (n = 3). *G*, cell growth curves were plotted using a cell counting assay (n = 4). *H*, colony-forming abilities were evaluated by a colony formation assay (n = 3, (∗∗∗∗) *p* < 0.0001 vs. shE2F7 + tet shNC group, #*p* < 0.05 vs. shE2F7 + tet shMYBL2 group). *I*, cell cycle distributions were assessed by flow cytometry (n = 3, (∗∗) *p* < 0.01/(∗∗∗) *p* < 0.001 vs. shE2F7 + tet shNC group). *J*, cell proliferation levels were assessed by EdU immunofluorescence assay (n = 3, (∗∗) *p* < 0.01 vs. shE2F7 + tet shNC group). Scale bar: 100 μm.

**Supplementary Figure S4:**
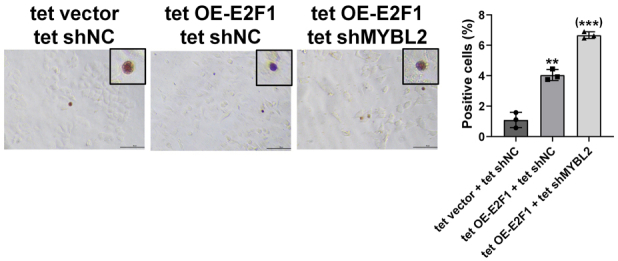


Our data revealed that knockdown of E2F1 or MYBL2 inhibited the proliferation of GC cells and that E2F1 transcriptionally activated MYBL2. To determine whether E2F1 promoted GC cell proliferation by transcriptionally activating MYBL2, we overexpressed E2F1 in AGS cells and then knocked down MYBL2 upon E2F1 overexpression using a Tet-On system (Figs. 5*A*, S1*F*). Growth curves showed that cells overexpressing E2F1 grew faster than those in the controls, and cells overexpressing E2F1 with MYBL2 knockdown grew slower than those with E2F1 overexpression alone but faster than those with MYBL2 knockdown alone (Fig. 5*B*). Similarly, a colony formation assay showed that the E2F1-overexpressing group had more colonies than the control group, but MYBL2 knockdown attenuated the effect of E2F1 overexpression, whereas the MYBL2-knockdown group had the fewest colonies (Fig. 5*C*). Importantly, MYBL2 knockdown effectively attenuated the effects of E2F1 overexpression on promoting the cell cycle transition from G1 to S and G2/M phases (Fig. 5*D*), increasing the percentage of EdU-positive cells (Fig. 5*E*) and increasing the Ki67 and PCNA protein levels (Figs. 5*A*, S1*F*). Taken together, these data suggest that E2F1 promotes GC cell proliferation, at least in part, by transcriptionally activating MYBL2. Interestingly, MYBL2 knockdown failed to attenuate the proapoptotic effect of E2F1 overexpression (Fig. S4), suggesting that E2F1 promotes apoptosis independent of its transcriptional activation of MYBL2.

Similarly, we also knocked down E2F7 in AGS cells in the presence or absence of MYBL2 knockdown (Figs. 5*F*, S1*G*). Compared with control cells, cells with E2F7 knockdown grew faster, whereas cells with E2F7 and MYBL2 double knockdown grew slower than those with E2F7 knockdown alone (Fig. 5*G*). Additionally, although the E2F7-knockdown group had more colonies than the control group, double-knockdown attenuated this effect (Fig. 5*H*). Furthermore, MYBL2 knockdown effectively attenuated the effects of E2F7 knockdown on promoting the G1‒S transition (Fig. 5*I*), increasing the percentage of EdU-positive cells (Fig. 5*J*) and increasing the Ki67 and PCNA protein levels (Figs. 5*F*, S1*G*). These data strongly suggest that E2F7 inhibits the proliferation of GC cells, at least in part, by transcriptionally repressing MYBL2.

**Figure 6:**
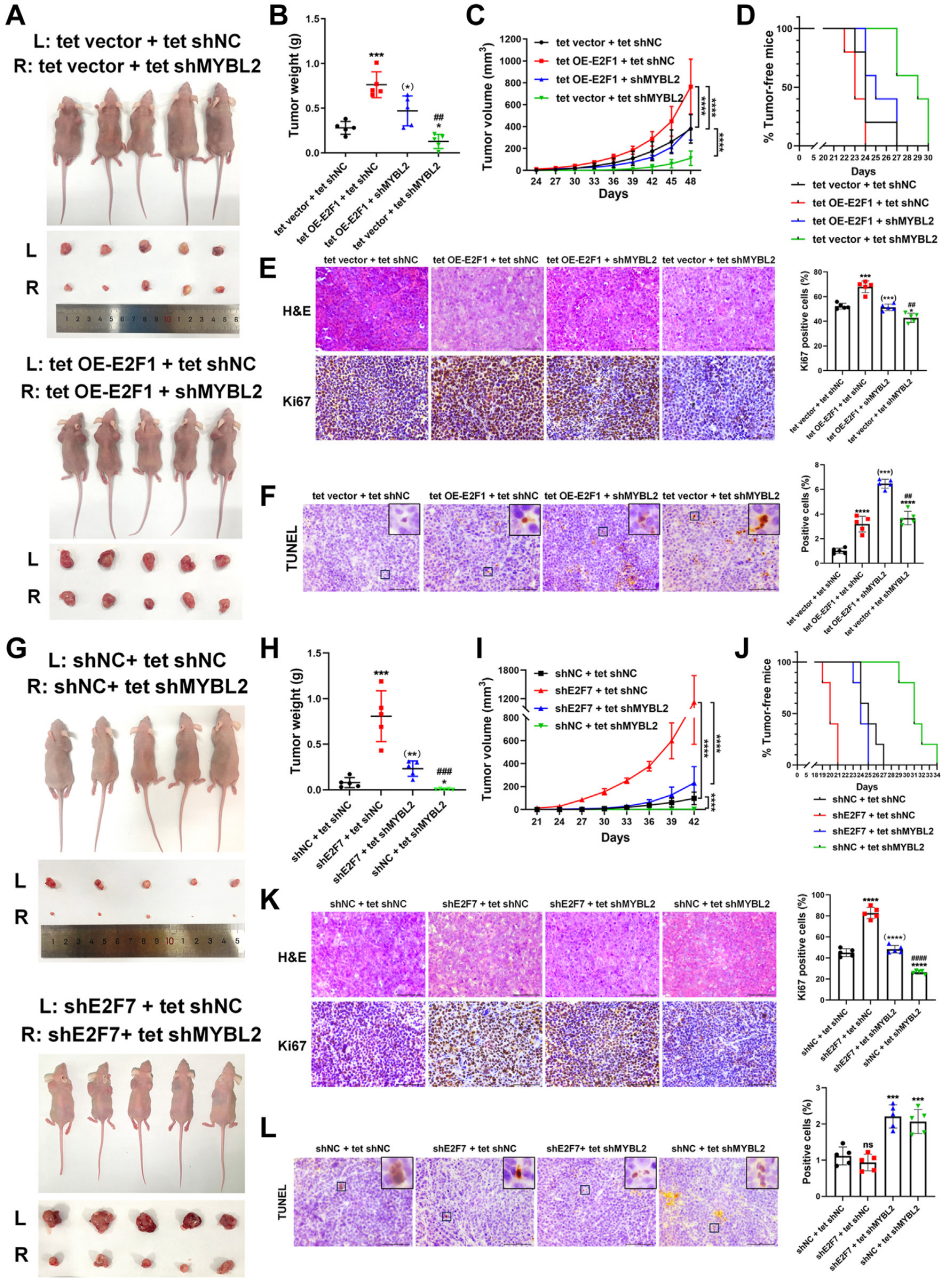
**Knockdown of MYBL2 attenuates the tumor-promoting effects of E2F1 overexpression or E2F7 knockdown *ex vivo*.***A*, representative images of nude mice and tumors stripped from mice after the injection of AGS cells of four groups induced by doxycycline: tet vector + tet shNC, tet OE-E2F1 + tet shNC, tet OE-E2F1 + shMYBL2 and tet vector + tet shMYBL2 (n = 5). L: *left*. R: *right*. *B*, tumor weights of xenograft tumors in nude mice (n = 5). *C*, growth curves of tumor xenografts in nude mice (n = 5). *D*, Kaplan-Meier tumor-free curves in different groups of nude mice were presented (n = 5). *E*, representative images of *H* & *E* staining and Ki67 IHC staining in tumor samples. Scale bar: 50 μm. The percentages of positive cells were shown in the *right* panel (n = 5, (∗∗) *p* < 0.01 vs. tet OE-E2F1 + tet shNC group, #*p* < 0.05 vs. tet OE-E2F1 + tet shMYBL2 group). *F*, apoptosis of tumor samples was assessed by a TUNEL assay. Scale bar: 50 μm. The percentages of positive cells were shown in the right panel (n = 5, (∗∗∗∗) *p* < 0.0001 vs. tet OE-E2F1 + tet shNC group, ##*p* < 0.01 vs. tet OE-E2F1 + tet shMYBL2 group). *G*, representative images of nude mice and tumors stripped from mice after the injection of AGS cells of four groups induced by doxycycline: shNC + tet shNC, shE2F7 + tet shNC and shE2F7 + tet shMYBL2 and shNC + tet shMYBL2 (n = 5). L: *left*. R: *right*. *H*, tumor weights of tumor xenografts in (*G*) (n = 5). *I*, growth curves of tumor xenografts in (*G*) (n = 5). *J*, Kaplan-Meier tumor-free curves in different groups of nude mice (*G*) were presented (n = 5). *K*, representative images of *H* & *E* staining and Ki67 IHC staining in tumor samples in (*G*). Scale bar: 50 μm. The percentages of positive cells were shown in the right panel (n = 5, (∗∗∗∗) *p* < 0.0001 vs. shNC + tet shNC group, ####*p* < 0.0001 vs. shE2F7 + tet shMYBL2 group). *L*, apoptosis of tumor samples in (*G*) was assessed by a TUNEL assay. Scale bar: 50 μm. The percentages of positive cells were shown in the *right* panel (n = 5).

To evaluate the effects of MYBL2 knockdown on E2F1 overexpression or E2F7 knockdown in a more physiologically relevant setting, we performed tumor xenograft assays using genetically modified AGS sublines generated using the Tet-On system. Although E2F1 overexpression significantly increased tumor volume, tumor weight, and tumor growth, these effects were attenuated by MYBL2 knockdown (Fig. 6, *A*–*C*). Kaplan‒Meier tumor-free curves showed that although the MYBL2-knockdown group presented the latest tumor onset, the mice with E2F1 overexpression and MYBL2 knockdown appeared to have later tumor onset than those with E2F1 overexpression alone (Fig. 6*D*). In addition, IHC staining of Ki67 showed that the E2F1-overexpressing group had more proliferative cells than the control group, whereas the MYBL2-knockdown group with E2F1 overexpression had fewer proliferative cells than the E2F1-overexpressing group (Fig. 6*E*). TUNEL assays showed that the percentage of TUNEL-positive cells was higher in the E2F1-overexpressing group and the MYBL2-knockdown group than in the control group, but was the highest in the MYBL2-knockdown group with E2F1 overexpression (Fig. 6*F*).

The biological functions of E2F7 and MYBL2 were also evaluated *ex vivo*. E2F7 knockdown led to increases in tumor volume, tumor weight, tumor growth, Ki67-positive cells, and earlier tumor onset, all of which were attenuated by MYBL2 knockdown (Fig. 6, *G*–*K*). Consistent with the *in vitro* data (Fig. 2*H*), E2F7 knockdown had no effect on apoptosis in tumor xenografts, but MYBL2 knockdown promoted apoptosis (Fig. 6*L*). Taken together, the data from the tumor xenografts support that E2F1 and E2F7 transcriptionally activate and repress MYBL2 to promote and inhibit GC cell proliferation, respectively.

**Supplementary Figure S5:**
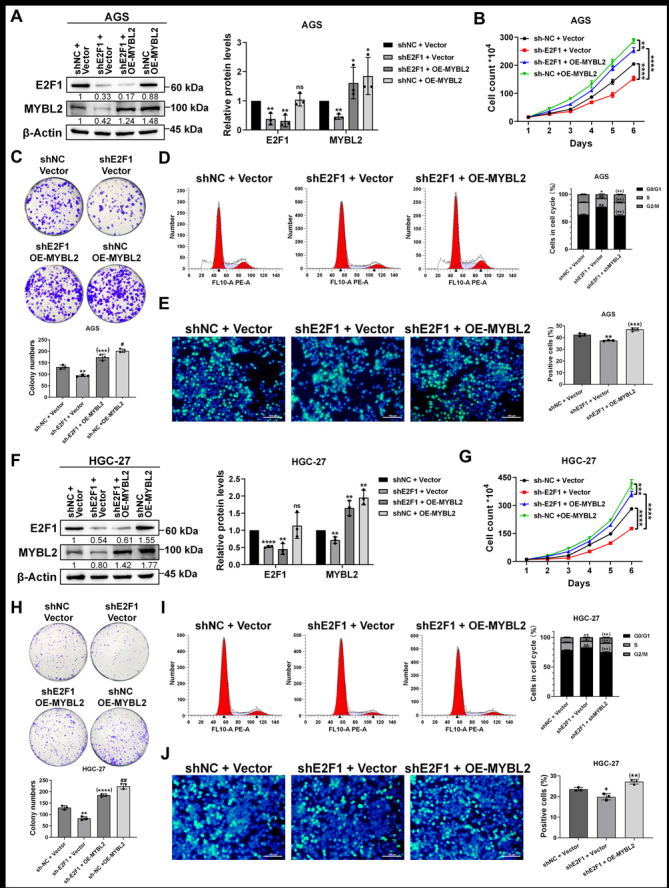


To further support that E2F1 and E2F7 affect cell growth and proliferation by their transcriptional regulation of MYBL2, we overexpressed MYBL2 in AGS cells upon efficient E2F1 knockdown (Fig. S5*A*) to determine whether MYBL2 overexpression reverses the cell growth inhibitory effects of E2F1 knockdown. Growth curves showed that E2F1-knockdown cells grew much slower than control cells, but overexpression of MYBL2 completely reversed the inhibitory effect on cell growth by E2F1-knockdown (Fig. S5*B*). Similarly, a colony formation assay showed that although the E2F1-knockdown group had fewer colonies than the control group, MYBL2 overexpression attenuated the inhibitory effect of E2F1 knockdown on colony formation (Fig. S5*C*). It is worth noting in both growth curve assays and colony formation assays, E2F1 knockdown had small but statistically significant inhibitory effects on MYBL2-overexpression-mediated enhancement of cell growth and colony formation. Importantly, MYBL2 overexpression effectively attenuated the effects of E2F1 knockdown on blocking the cell cycle transition from the G1 to the S and G2/M phases (Fig. S5*D*) and on decreasing the percentage of EdU-positive cells (Fig. S5*E*). These data demonstrated that overexpression of MYBL2 promoted the proliferation of GC cells under conditions of efficient E2F1 knockdown, which was also verified in HGC-27 cells (Fig. S5, *F*–*J*). Therefore, we conclude that inhibition of cell growth and proliferation by E2F1 knockdown can be rescued by MYBL2 overexpression, further supporting that MYBL2 is a key functional target of E2F1 in GC cells.

**Supplementary Figure S6:**
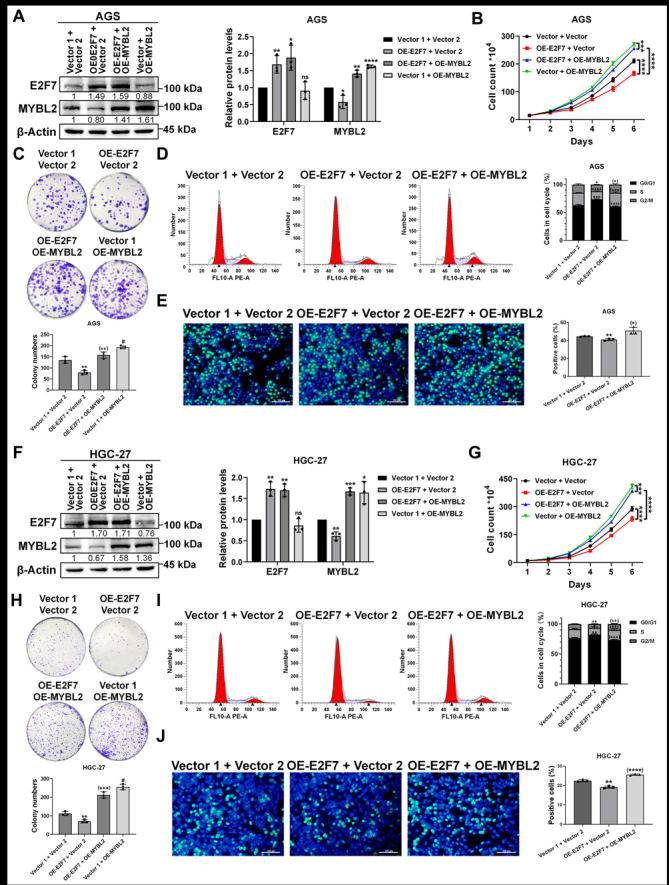


In parallel, we also overexpressed E2F7 in AGS and HGC-27 cells with or without MYBL2 overexpression (Fig. S6, *A* and *F*). Compared with control cells, cells with E2F7 overexpression grew more slowly, but overexpression of MYBL2 completely reversed the growth inhibitory effect of E2F7 overexpression as cells with E2F7 and MYBL2 double overexpression grew faster than those with E2F7 overexpression alone (but slightly slower than those with MYBL2 overexpression alone) (Fig. S6, *B* and *G*). Additionally, the E2F7 overexpression group had fewer colonies than the control group, but double overexpression attenuated this effect, while the group with MYBL2 overexpression alone had the most colonies (Fig. S6, *C* and *H*). Furthermore, MYBL2 overexpression effectively attenuated the inhibitory effect of E2F7 overexpression on the cell cycle transition from the G1 to the S and G2/M phases (Fig. S6, *D* and *I*) and on the percentage of EdU-positive cells (Fig. S6, *E* and *J*). Taken together, the inhibition of cell growth and proliferation by E2F7 overexpression can be rescued by MYBL2 overexpression, further supporting that MYBL2 is also a key functional target of E2F7 in GC cells.

### PI3K and AKT are important effectors of E2F1- and E2F7-mediated transcriptional regulation of MYBL2 in GC cells

**Figure 7:**
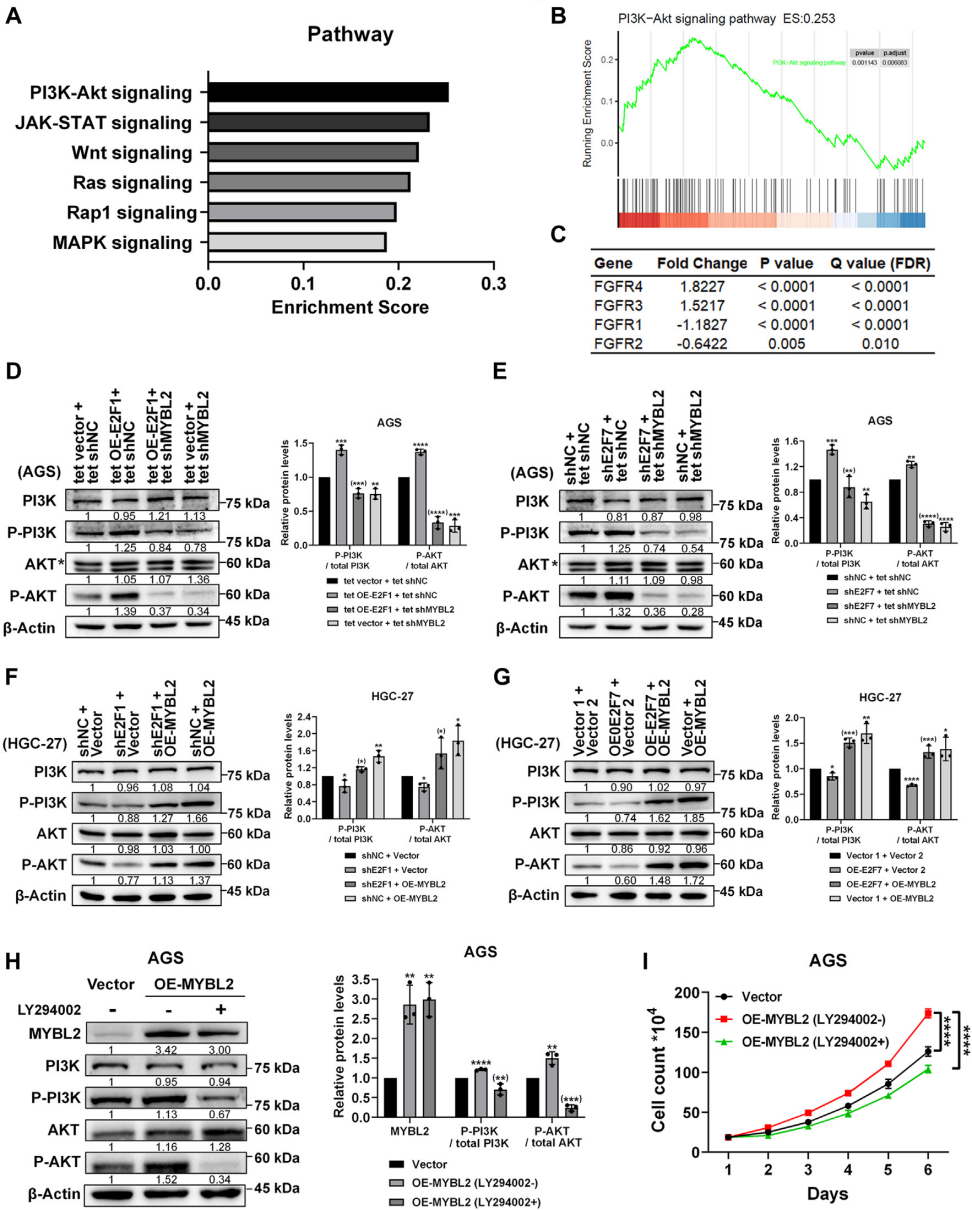
**E2F1 and E2F7 activate and repress MYBL2 to regulate cell growth through the PI3K/AKT pathway.***A*, oncogenic signaling pathways enriched by GSEA based on the differentially expressed genes between GC tissues with high MYBL2 expression levels and those with low MYBL2 expression levels. *B*, GSEA plot showing that the MYBL2-regulated genes correlate with the PI3K/AKT signaling pathway. *C*, the FGFR family genes among differentially expressed genes. *D*, PI3K, P-PI3K, AKT and P-AKT protein levels in tet vector + tet shNC, tet OE-E2F1 + tet shNC, tet OE-E2F1 + shMYBL2, and tet vector + tet shMYBL2 groups of AGS cells were evaluated by WB (n = 3, (∗∗∗) *p* < 0.001/(∗∗∗∗) *p* < 0.0001 vs. tet OE-E2F1 + tet shNC group). *E*, PI3K, P-PI3K, AKT and P-AKT protein levels in shNC + tet shNC, shE2F7 + tet shNC, shE2F7 + tet shMYBL2, and shNC + tet shMYBL2 groups of AGS cells were evaluated by WB (n = 3, (∗∗) *p* < 0.01/(∗∗∗∗) *p* < 0.0001 vs. shE2F7 + tet shNC group). *F*, PI3K, P-PI3K, AKT and P-AKT protein levels in shNC + Vector, shE2F1 + Vector, shE2F1 + OE-MYBL2, and shNC + OE-MYBL2 groups of HGC-27 cells were evaluated by WB (n = 3, (∗) *p* < 0.05 vs. shE2F1 + Vector group). *G*, PI3K, P-PI3K, AKT and P-AKT protein levels in Vector 1 + Vector 2, OE-E2F7 + Vector 2, OE-E2F7 + OE-MYBL2, and Vector 1 + OE-MYBL2 groups of HGC-27 cells were evaluated by WB (n = 3, (∗∗∗) *p* < 0.001 vs. OE-E2F7 + Vector two group). *H*, changes in MYBL2, PI3K, P-PI3K, AKT and P-AKT protein levels after MYBL2 overexpression treated with or without LY294002 in AGS cells were evaluated by WB (n = 3). *I*, changes in cell growth curves drawn by cell counting after MYBL2 overexpression treated with or without LY294002 in AGS cells (n = 4).

To further explore the mechanism by which E2F1 and E2F7 regulate GC cell growth and proliferation by transcriptionally modulating MYBL2, we analyzed RNA sequencing data from primary GC samples in the TCGA database. After the samples were categorized based on low and high expression of MYBL2, we obtained a list of differentially expressed genes and conducted gene set enrichment analysis (GSEA) to determine the correlation between MYBL2 and oncogenic pathways. The results showed that the dysregulated genes were involved in multiple cancer-related pathways, such as phosphatidylinositol 3-kinase (PI3K)/protein kinase B (AKT) signaling, Janus kinase (JAK)/signal transducer and activator of transcription (STAT) signaling, and Wnt signaling, *etc.* (Fig. 7*A*), with the PI3K/AKT pathway being the most significantly enriched (Fig. 7, *A* and *B*). Among the upstream kinases that can activate the PI3K/AKT signaling pathway, fibroblast growth factor receptors (FGFRs) are present on the list of differentially expressed genes (Fig. 7*C*). Specifically, FGFR3 and FGFR4 are significantly upregulated in the MYBL2 high-expression group compared to the MYBL2 low-expression group (Fig. 7*C*). Considering that MYBL2 has been shown to activate the PI3K/AKT pathway in colorectal cancer cells (45) and non-small cell lung cancer cells (46) and that E2F1 activated the PI3K/AKT pathway in squamous cell carcinoma cells (47, 48), we determined whether the PI3K/AKT pathway can mediate the function of E2F1, E2F7, and MYBL2 in GC cells.

We first utilized WB to assess the changes in protein levels of total PI3K, phosphorylated PI3K (P-PI3K), total AKT, and phosphorylated AKT (P-AKT) in GC cells with various genetic manipulations of E2F1, E2F7, and MYBL2. As shown in Figure 7*D*, in AGS cells, although overexpression of E2F1 did not change protein levels of total AKT or total PI3K, it elevated protein levels of P-PI3K and P-AKT, effects that were reversed by MYBL2 knockdown. Similarly, the knockdown of E2F7 elevated protein levels of P-PI3K and P-AKT, effects that were reversed by MYBL2 knockdown (Fig. 7*E*). In addition, in HGC-27 cells, the knockdown of E2F1 decreased the protein levels of P-PI3K and P-AKT, effects that were reversed by MYBL2 overexpression (Fig. 7*F*). Similarly, overexpression of E2F7 decreased the protein levels of P-PI3K and P-AKT, effects that were reversed by MYBL2 overexpression (Fig. 7*G*). These data suggest that PI3K and AKT are important effectors of the E2F1/E2F7-MYBL2 signaling axis in GC cells. Based on the observed changes in the activation status of PI3K and AKT, we conclude that E2F1 and MYBL2 activate while E2F7 suppresses the PI3K/AKT pathway, and the regulatory effects of E2F1 and E2F7 on the PI3K/AKT signaling pathway in GC cells are at least partially mediated through the modulation of MYBL2 expression.

To determine whether PI3K and AKT are involved in the E2F1/E2F7-MYBL2 signaling axis in GC cells, we next overexpressed MYBL2 in AGS cells and treated them with the PI3K inhibitor LY294002. WB revealed that as expected, in MYBL2-overexpressing cells not treated with LY294002, protein levels of both P-PI3K and P-AKT were greater than those in the control group (Fig. 7*H*). However, treatment of LY294002 in MYBL2-overexpressing cells reversed the effects of MYBL2-overexpression on elevating protein levels of P-AKT and P-PI3K (to a lesser extent) (Fig. 7*H*). Importantly, growth curve analysis showed that although MYBL2-overexpressing cells grew faster than control cells, treatment of LY294002 in MYBL2-overexpressing cells reversed the effects of MYBL2 on promoting cell growth (Fig. 7*I*), indicating that inhibition of the PI3K/AKT pathway can attenuate the proliferative effect of MYBL2 in GC cells. Taken together, these data strongly suggest that the effects of E2F1 and E2F7 on GC cell proliferation through transcriptional regulation of MYBL2 are mediated, at least in part, by the PI3K/AKT signaling pathway.

### Differential nucleocytoplasmic distribution of E2F7 in GC cells has functional relevance

**Figure 8:**
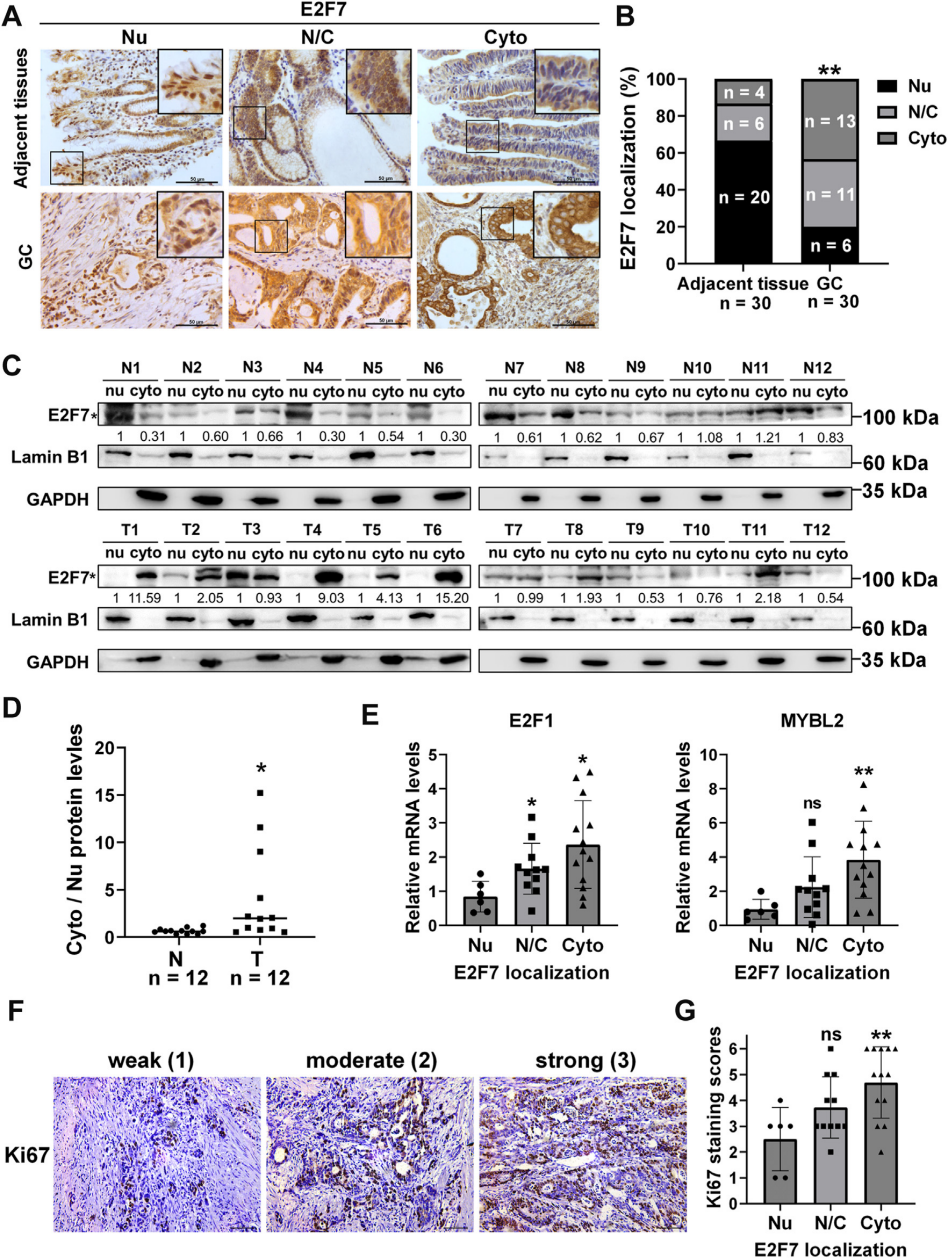
**Higher levels of nuclear E2F7 protein in GC cells correspond to lower mRNA levels of E2F1 and MYBL2 and lower protein levels of Ki67.***A*, nucleocytoplasmic distributions of E2F7 protein in adjacent tissues and GC tissues were evaluated by IHC staining (the N/C/GC panel reuses the *bottom left* quadrant from Fig. 1*C* to highlight the specific distribution pattern). Scale bar: 50 μm. *B*, percentage of different E2F7 localization in adjacent tissues and GC tissues (λ^2^ test, *p* < 0.01). *C*, nucleocytoplasmic distributions of E2F7 protein in 12 paired adjacent tissues and GC tissues were evaluated by WB. “∗” represented the specific band. *D*, statistics of the ratios of cytoplasmic E2F7 protein levels to nuclear E2F7 protein levels in (*C*). *E*, mRNA levels of E2F1 and MYBL2 in 30 GC samples with different nucleocytoplasmic distributions of E2F7 protein. *F*, representative images of IHC for Ki67 with different staining scores in GC tissues. Scale bars: 50 μm. *G*, statistics of Ki67 IHC staining scores in 30 GC samples with different nucleocytoplasmic distributions of E2F7 protein.

To date, several lines of seemingly contradictory data have drawn our attention. *In vitro* experiments suggested that E2F7 plays a tumor suppressor role in GC cells (Fig. 2, *C*–*G*). However, the mRNA and protein levels of E2F7 were greater in GC tissues than in normal tissues (Fig. 1, *A*–*D*), suggesting that E2F7 may promote GC. In addition, although E2F7 transcriptionally repressed MYBL2 in GC cells (Fig. 4), the mRNA levels of E2F7 and MYBL2 were positively correlated in GC tissues (Fig. 3, *A* and *B*). One potential explanation for these seemingly contradictory data is that in GC cells, much of the E2F7 protein is localized in the cytoplasm instead of the nucleus, attenuating its function as a transcriptional repressor or tumor suppressor. If this were the case, high levels of E2F7 in GC cells could result from compensatory upregulation for its reduced transcriptional activity due to its increased cytoplasmic distribution. Therefore, based on E2F7 protein localization assessed by IHC, we classified 30 GC samples into three types: primarily nuclear (Nu), nuclear and cytoplasmic (N/C), and primarily cytoplasmic (Cyto) (Fig. 8*A*). Compared with that in paracancerous cells, the cytoplasmic distribution of E2F7 in GC cells was significantly greater (Fig. 8*B*). We also evaluated E2F7 nucleocytoplasmic distributions in 12 paired GC and adjacent tissues by WB and found that E2F7 had greater cytoplasmic distributions in GC tissues than in paracancerous tissues (Fig. 8, *C* and *D*).

Next, we correlated the E2F7 nucleocytoplasmic localization groups assessed by IHC with the mRNA levels of E2F1 and MYBL2 and found that GC tissues with increased cytoplasmic distributions of E2F7 presented increased mRNA levels of E2F1 and MYBL2 (Fig. 8*E*). The estimation plots, which better visualize the mean difference between the two groups, are presented in Fig. S7, *A* and *B*. Importantly, IHC showed that samples with a cytoplasmic distribution of E2F7 had higher Ki67 protein levels than those with a nuclear distribution (Fig. 8, *F* and *G*). The estimation plot is presented in Fig. S7*C*. Taken together, these data suggest that the differential nucleocytoplasmic distribution of E2F7 in GC cells has functional relevance, as it is correlated with the role of E2F7 in repressing transcription and inhibiting cell proliferation.

**Figure 9:**
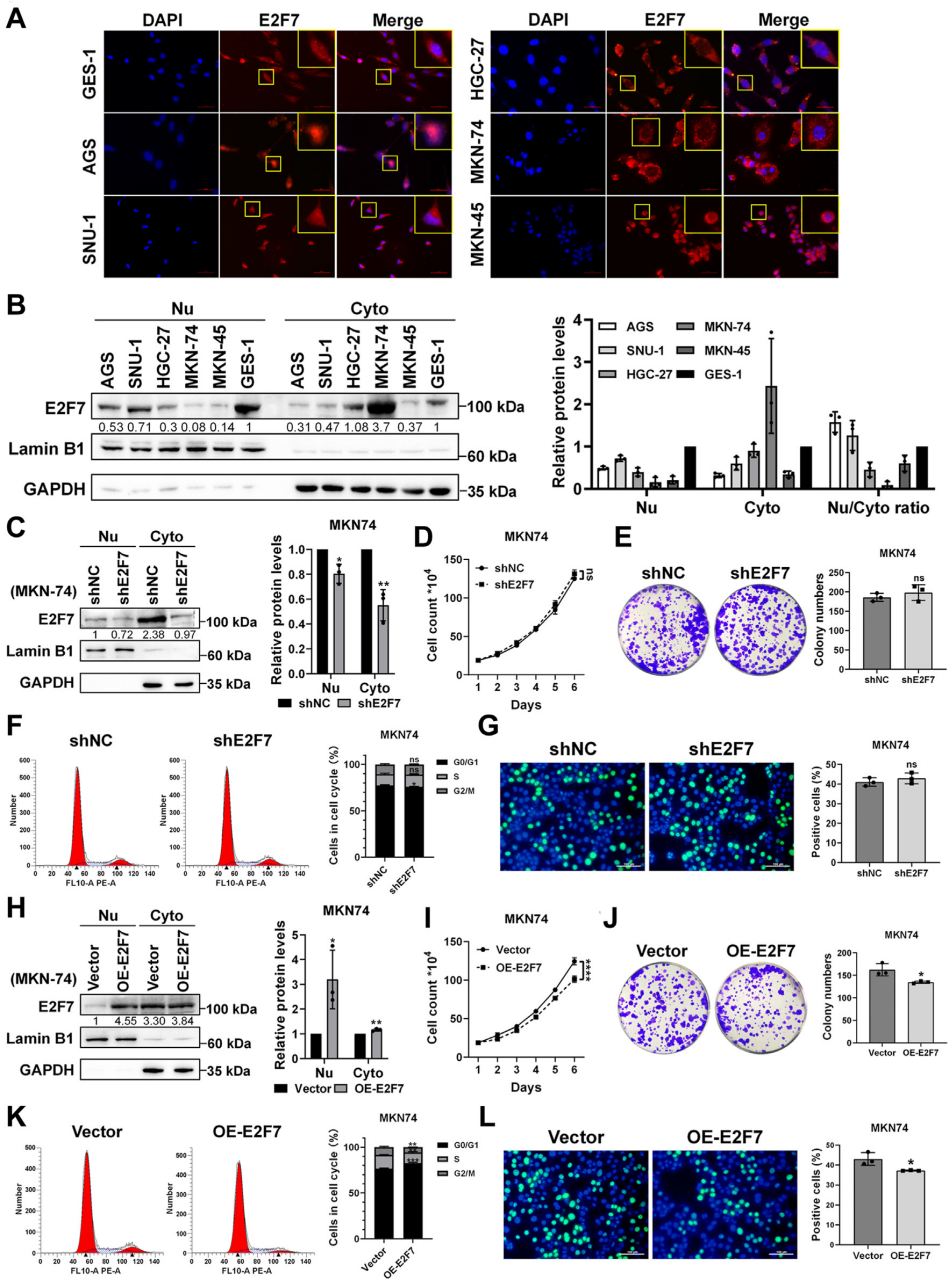
**The nuclear protein levels of E2F7 determine its effect on cell proliferation.***A*, nucleocytoplasmic localization of E2F7 protein in AGS, SNU-1, HGC-27, MKN74, MKN45, and GES-1 cells was evaluated by immunofluorescence. *B*, nucleocytoplasmic distribution of E2F7 protein in six cell lines were evaluated by WB (n = 3). *C*, changes in nuclear and cytoplasmic protein levels of E2F7 following knockdown in MKN74 cells were evaluated by WB (n = 3). *D*, changes in cell growth curves drawn by cell counting after E2F7 knockdown in MKN74 cells (n = 4). *E*, effects of E2F7 knockdown on the colony formation ability of MKN74 cells were assessed by a colony formation assay (n = 3). *F*, changes in cell cycle distributions after E2F7 knockdown in MKN74 cells were evaluated by flow cytometry (n = 3). *G*, changes in cellular proliferation of AGS cells after E2F7 knockdown were assessed by EdU immunofluorescence staining (n = 3). Scale bar: 100 μm. *H*, changes in nuclear and cytoplasmic protein levels of E2F7 following overexpression in MKN74 cells were evaluated by WB (n = 3). *I*, changes in cell growth curves drawn by cell counting after E2F7 overexpression in MKN74 cells (n = 4). *J*, effects of E2F7 overexpression on the colony formation ability of MKN74 cells were assessed by a colony formation assay (n = 3). *K*, changes in cell cycle distributions after E2F7 overexpression in MKN74 cells were evaluated by flow cytometry (n = 3). *L*, changes in cellular proliferation of AGS cells after E2F7 overexpression were assessed by EdU immunofluorescence staining (n = 3). Scale bar: 100 μm.

Interestingly, our data from the aforementioned experiments indicated that E2F7 knockdown significantly promoted cell proliferation in both AGS and HGC-27 cells. This observation prompted us to assess the relative nuclear‒cytoplasmic distribution of the E2F7 protein in AGS and HGC-27 cells. First, we assessed the nuclear‒cytoplasmic distribution of E2F7 in 5 GC cell lines and the normal gastric mucosal cell line GES-1 using immunofluorescence. As shown in Fig. 9*A*, E2F7 was predominantly nuclear in AGS and SNU-1 cells, but primarily cytoplasmic in HGC-27, MKN74, and MKN45 cells. We then performed WB to assess the protein levels of both nuclear and cytoplasmic E2F7 in these cell lines. Among the GC cell lines, AGS cells presented the highest nuclear‒cytoplasmic ratio and relatively high nuclear protein levels of E2F7 (Fig. 9*B*), which may account for the potent growth-promoting effect observed upon E2F7 knockdown in AGS cells. In HGC-27 cells, although E2F7 was slightly more abundant in the cytoplasm, its nuclear protein levels were not low (Fig. 9*B*), which may explain why the knockdown of E2F7 in HGC-27 cells also had a strong growth-promoting effect. Notably, despite the high total protein levels of E2F7 in MKN74 cells (Fig. 2*A*), both the nuclear‒cytoplasmic distribution ratio and the nuclear protein levels were the lowest (Fig. 9*B*). Consequently, we knocked down E2F7 in MKN74 cells and assessed the changes in nuclear and cytoplasmic E2F7 protein levels by WB. Although E2F7 knockdown reduced cytoplasmic E2F7 protein levels by about 50%, it only reduced nuclear E2F7 protein levels by about 20% (Fig. 9*C*). Data from a series of experiments including growth curve assay by cell counting, colony formation, cell cycle distribution analysis by flow cytometry, and EdU immunofluorescence showed that knockdown of E2F7 in MKN74 cells had no significant effects on cell growth or cell proliferation (Fig. 9, *D–G*). In contrast, overexpression of E2F7 in MKN74 cells substantially elevated the nuclear protein levels of E2F7 with little effect on the cytoplasmic protein levels (Fig. 9*H*), and significantly inhibited cell growth and cell proliferation (Fig. 9, *I*–*L*). These data suggest that the nuclear protein levels of E2F7, rather than the total protein levels, are the determinants of GC cell proliferation.

## Discussion

**Figure 10:**
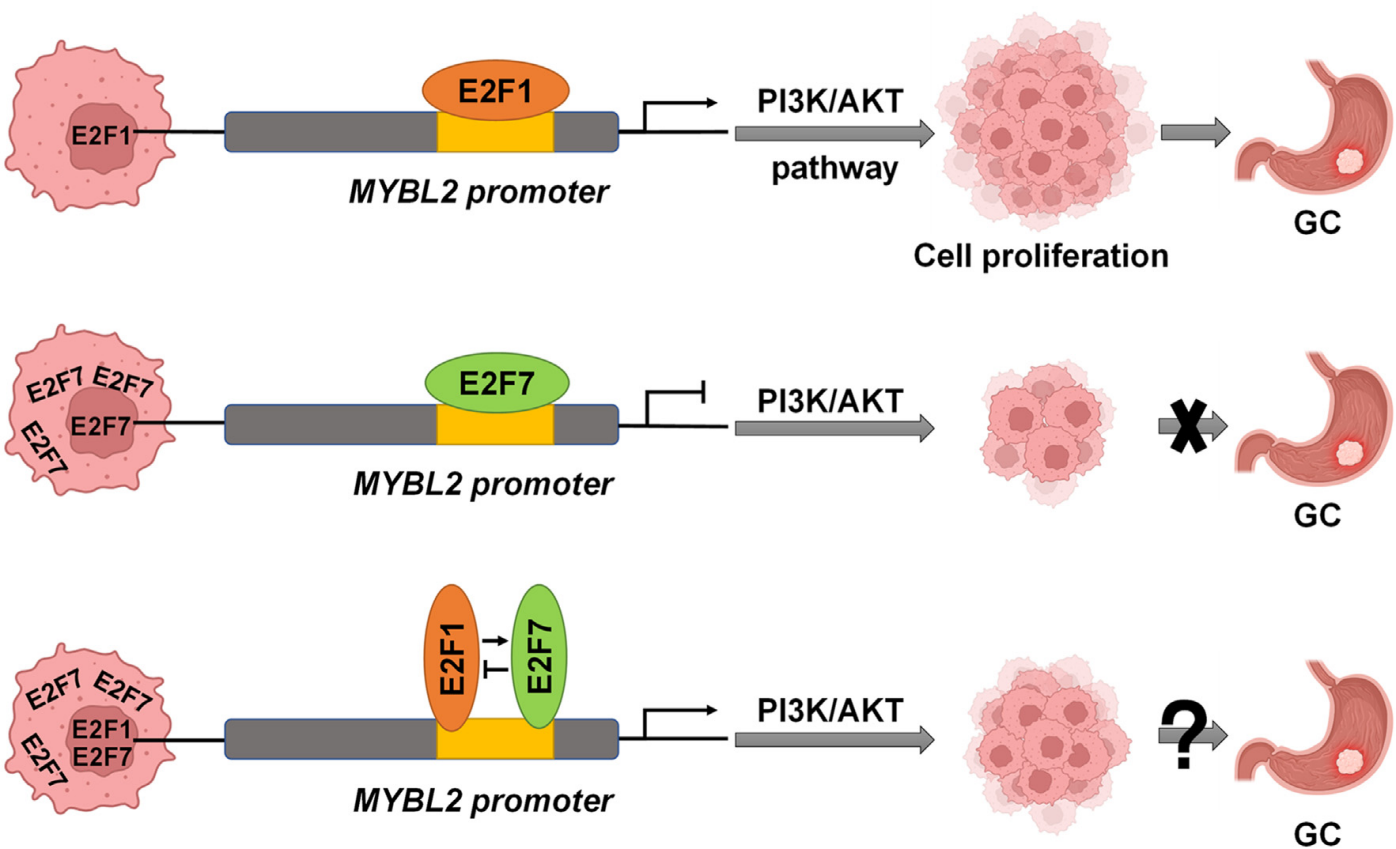
**Work model.** E2F1 and E2F7 promote or inhibit cell proliferation in GC cells through direct transcriptional activation or repression of MYBL2 *via* PI3K/AKT signaling pathway, and form a negative feedback regulation in which E2F1 up-regulates E2F7 and E2F7 down-regulates E2F1. The nuclear protein levels of E2F7 determine the extent of its transcriptional repression and its impact on cell proliferation.

Although surgery, systemic chemotherapy, radiotherapy, immunotherapy, and targeted therapy have been widely used to treat GC (49, 50, 51), antitumor drugs targeting the upstream kinases of the RB-E2F pathway are currently only in clinical trials for GC, and there are no drugs directly targeting E2F that are currently in clinical trials (52). Here, we combined both bioinformatic analysis and empirical studies using clinical specimens and cell culture systems to empirically establish a tumor suppressive role of transcriptional repressor E2F7 and to better define an oncogenic role of transcriptional activator E2F1 and their potential target gene MYBL2 in GC. In addition, we used cellular experiments and a xenograft mouse model to uncover that MYBL2 is a functionally relevant target of E2F1 and E2F7 in GC cells as both E2Fs affected the proliferation of GC cells through transcriptional regulation of MYBL2. Furthermore, we identified that the PI3K/AKT signaling pathway was an important effector for E2F-dependent transcriptional regulation of MYBL2. Interestingly, differential nucleocytoplasmic distribution of E2F7 is not only present in GC cells but also has functional relevance. Overall, our data support that E2F1 and E2F7 modulate GC cell proliferation through direct transcriptional regulation of MYBL2 to impact the PI3K/AKT signaling pathway (Fig. 10).

As discussed earlier, in addition to confirming an oncogenic effect of E2F1 and MYBL2 in GC cells (Fig. 2), our study represents the first demonstration that E2F1 transcriptionally activates MYBL2 in GC cells (Fig. 4), and that the oncogenic effect of E2F1 on GC cells is dependent, at least in part, on its transcriptional activation of MYBL2 (Figs. 5 and 6, S5 and S6). While the direct effects of E2F7 on proliferation and apoptosis have not been previously reported in GC cells, our study fills such knowledge gap by showing that knockdown of E2F7 in GC cells promoted cell proliferation but had no obvious effect on apoptosis (Fig. 2). Importantly, our data demonstrated for the first time that E2F7 transcriptionally repressed MYBL2 in GC cells (Fig. 4) and that the tumor suppressive role of E2F7 on GC cells was partly dependent on its transcriptional repression of MYBL2 (Figs. 5 and 6, S5 and S6). Mechanistically, we demonstrated that the positive effects on cell growth and cell proliferation by E2F1 overexpression or E2F7 knockdown can be rescued by MYBL2 knockdown (Figs. 5 and 6), whereas the negative impacts on cell growth and cell proliferation by E2F1 knockdown or E2F7 overexpression can be rescued by MYBL2 overexpression (Figs. S5 and S6). These data strongly support that MYBL2 is a key functionally relevant target of E2F1 and E2F7 in regulating GC cell growth and proliferation. It is worth noting that as transcription factors, E2F1 and E2F7 have numerous targets. It would be interesting to know whether other E2F targets can also mediate the function of E2F1 or E2F7 in GC cells. For example, it has been reported that E2F1 can promote the proliferation of GC cells by transcriptionally activating topoisomerase II alpha (53) and that E2F7 can inhibit cell proliferation by transcriptionally regulating C-MYC, LIN28B, and numerous microRNAs (54).

It is well established that dysregulation of the PI3K/AKT pathway drives the occurrence and progression of cancer (55). Growth factor-mediated induction of receptor tyrosine kinases can trigger the activation of the PI3K/AKT signaling pathway (55). The main members of receptor tyrosine kinases include the epidermal growth factor receptors, vascular endothelial growth factor receptors, and FGFRs (56). Several studies have reported the activation of the PI3K/AKT signaling pathway by E2Fs in various human cancers, such as colorectal cancer (45), cervical squamous cell carcinoma (47), esophageal squamous cell carcinoma (48), and glioma (57). In addition, MYBL2 has also been reported to activate the PI3K/AKT pathway in colorectal cancer cells (45) and non-small cell lung cancer cells (46). In our study, we found that the high expression of MYBL2 in GC tissues correlated with the activation of the PI3K/AKT pathway (Fig. 7, *A* and *B*), and subsequently confirmed that the role of E2F1 and E2F7 in regulating GC cell proliferation by transcriptionally regulating MYBL2 can be mediated by the PI3K/AKT signaling pathway (Fig. 7, *D*–*I*). Interestingly, high expression of MYBL2 in GC tissues led to the upregulation of FGFR3 and/or FGFR4 (Fig. 7*C*). Whether MYBL2 activates the PI3K/AKT signaling pathway by upregulating members of the FGFR family warrants further investigation.

The reciprocal regulation of E2F1 and E2F7 in GC cells is of interest. There may be a complex balance between transcriptional activator E2F1 and transcriptional repressor E2F7 in GC cells and human bodies, with E2F1 playing a dominant role in some cases and E2F7 in others. Our data suggest that E2F1 and E2F7 play opposite roles in GC cells (Fig. 2, *C*–*H*). However, their mRNA levels were positively correlated in GC tissues (Fig. 3, *A* and *B*). In addition, in GC cells, E2F1 upregulated E2F7, whereas E2F7 downregulated E2F1 (Fig. 3, *C*–*H*), suggesting that E2F1 and E2F7 form a negative feedback loop to maintain a normal balance between the two E2F activities with opposite functions, thus ensuring homeostasis in normal cells. Therefore, disrupting the balance between them, by either transcriptional dysregulation or increased cytoplasmic distribution of E2F7, may lead to cancer development. Consistent with this speculation, in GC samples, high ratios of E2F1/E2F7 mRNA levels correlated with poor prognosis, whereas high ratios of E2F7/E2F1 mRNA levels correlated with better prognosis (Fig. 1*J*). There is evidence that both E2F1 and E2F7 bind to the E2F1 promoter in 293 cells (27). In breast cancer cells, E2F7 competes with E2F1 for binding to the promoter of its target gene, deleted in lymphocytic leukemia 2 (DLEU2) (58). Notably, our study demonstrated that in GC cells, both E2F1 and E2F7 bound to the same consensus E2F binding sites on the E2F1 promoter and the MYBL2 promoter (Fig. 4). Whether E2F1 and E2F7 competitively bind to the MYBL2 promoter and whether disrupting the balance between the two opposing E2F transcriptional activities influences the transcription of MYBL2 and consequently the development of GC is worth exploring further.

Previous studies have shown that the E2F7 protein has a cytoplasmic distribution in 80% of head and neck squamous cell carcinomas but in less than 10% of normal tissues (59). While at least 70% of prostate cancer, colon cancer, and breast cancer samples presented a cytoplasmic distribution of E2F7, fewer than 30% of corresponding paracancerous tissues presented a cytoplasmic distribution (59), suggesting that the cytoplasmic distribution of E2F7 may be an important mediator of cancer development. Consistent with this notion, our IHC and WB analyses showed a greater E2F7 cytoplasmic distribution in GC cells than in paracancerous cells (Fig. 8, *A*–*D*). In addition, we also showed that differential nucleocytoplasmic distribution of E2F7 in GC cells was correlated with the role of E2F7 in repressing transcription and inhibiting proliferation (Fig. 8, *E*–*G*). Furthermore, the nuclear protein levels of E2F7, rather than the total E2F7 protein levels, are the decisive factor in its role in inhibiting cell proliferation (Fig. 9). Although the scientific and clinical relevance of these findings require both the analysis of more clinical specimens and the exploration of the mechanism underlying E2F7 nucleocytoplasmic shuttling, such efforts may eventually lead to the development of novel targeted therapies for GC. Interestingly, anthracycline resistance in head and neck squamous cell carcinoma can be reversed by inhibiting the exportin 1-dependent nuclear export of E2F7 (59). In addition, inhibition of exportin one led to E2F7 nuclear accumulation, blocking neuroblastoma progression (60). In gallbladder cancer cells, karyopherin α2 increased the nuclear localization of E2F7 to promote E2F7-mediated transcriptional repression (61). These data suggest that critical molecules or signaling pathways responsible for the nuclear localization of E2F7 are worthy of further investigation and may be promising candidates for targeted therapies in GC. Overall, our data not only provide important insights into the roles and mechanisms of E2F1, E2F7 and MYBL2 in GC but also shed light on potential targeted therapies for GC from three different angles: E2F1, E2F7 and MYBL2 themselves, E2F1/E2F7 expression ratios, and E2F7 nuclear localization.

## Experimental procedures

### Patients, specimens, and cell lines

We collected 30 pairs of specimens from Liaoning Cancer Hospital and Institute between 2017 and 2018, with each pair consisting of GC tissues and their adjacent non-tumor tissues. The histology of all specimens was verified by resident pathologists at the hospital. The study was conducted in accordance with the Declaration of Helsinki, and approved by the Ethics Committee of Cancer Hospital of China Medical University (protocol code: 2021G0337). All patients provided informed consent.

The 293T/17 cell line was purchased from Shanghai Fuheng Biotechnology Co., Ltd. AGS, HGC-27, MKN-45, and GES-1 cell lines were gifts from Dr Jie Lv at China Medical University. SNU-1 and MKN-74 cell lines were gifts from Dr Wanchuan Zhang at the Liaoning Cancer Hospital and Institute. Cell lines were tested by short tandem repeat analysis and tested for *mycoplasma*. 293T/17 cells were cultured in DMEM medium (12800017, Gibco) supplemented with 10% fetal bovine serum (FBS) (FB25015, CLARK). All other cells were cultured in 1640 medium (31800022, Gibco) with 10% FBS. All cells were maintained at 37 °C and 5% CO_2_. To inhibit the PI3K/AKT pathway, AGS cells overexpressing MYBL2 were treated with LY294002 (20 μM, GC15485, GLPBIO, USA).

### Bioinformatic analysis

Gene expression data was downloaded from The Cancer Genome Atlas (TCGA) database (http://cancergenome.nih.gov/), which included 341 GC samples and 30 normal samples. The Kaplan Meier-plotter online analysis tool (http://kmplot.com/analysis/) was used to draw survival curves. Gene Set Enrichment Analysis (GSEA) at gsea-msigdb.org was performed to identify significantly altered pathways using transcriptome sequencing data from primary GC tumor samples in the TCGA database. We utilized the quartiles of MYBL2 gene expression as the cutoff criteria, classifying samples within the 0 to 25% range as those with low expression of the MYBL2 gene, and those within the 75 to 100% range as those with high expression. Genes exhibiting expression differences (fold change) greater than 0.5 or less than −0.5, and with a false discovery rate exceeding 0.05 when comparing the high-expression group to the low-expression group, were identified as differentially expressed genes. These differentially expressed genes were then employed for GSEA.

### Reverse-transcription quantitative PCR (RT-qPCR)

Total RNA was extracted from clinical samples or cells with TRNzol reagent (DP424, TIANGEN) according to the manufacturer’s instructions. RT-qPCR was performed using SYBR master mix (420A, Takara) in a StepOne Plus system (Applied Biosystems) with GAPDH as a normalization control. Sequences of RT-qPCR primers were listed in Table S4.

### Protein isolation and Western blot (WB)

Total proteins of cells and tissues were extracted with RIPA lysis buffer. Tissue lysis and separation of the nuclear and cytoplasmic fractions were done using Minute Cytosolic and Nuclear Extraction Kit for Frozen/Fresh Tissues (NT-032, Invent Biotechnologies), according to the manufacturer’s instructions. Cell lysis and separation of the nuclear and cytoplasmic fractions were done using NE-PER Nuclear and Cytoplasmic Extraction Reagents (78833, Thermo Scientific), according to the manufacturer’s instructions. WB was performed using antibodies against E2F1 (NBP2-67899, Novus, 1: 3000), E2F7 (24489-1-AP, Proteintech, 1: 3000), MYBL2 (sc-390198, Santa Cruz, 1: 300), Ki67 (bs-23103R, Bioss, 1: 2000), PCNA (13110, CST, 1: 5000), β-actin (66009-1-IG, Proteintech, 1: 20000), PI3K (4257, CST, 1:1000), phospho-PI3K (4228, CST, 1:2000), AKT (4691, CST, 1:2000), phospho- AKT (4060, CST, USA, 1:2000), Lamin B1 (WL01775, Wanleibio, 1: 4000), and GAPDH (D190090, Sangon Biotech, 1: 5000). Anti-rabbit and anti-mouse secondary antibodies (TC262979 and UB280570, Invitrogen) were used with 1:10000 dilution. Quantification of band intensities was performed using ImageJ software.

### Immunohistochemistry (IHC) and scoring

Immunohistochemical staining was performed using a standard immunoperoxidase staining kit (SP-9001, ZSGB-Bio, China) according to the manufacturer’s instructions. An E2F1 monoclonal antibody (NBP2-67899, Novus), an E2F7 polyclonal antibody (ab56022, Abcam), a MYBL2 polyclonal antibody (sc-390198, Santa Cruz), and a Ki67 polyclonal antibody (550609, BD) were diluted at 1: 100, 1: 50, 1: 50, and 1: 200, respectively. Two investigators who were blind to the pathological status of each specimen assessed the staining independently. Samples were semi-quantitatively scored for the percentage of stained cells as follows: 0 for 0% immunoreactive cells; 1 for < 10% immunoreactive cells; 2 for 11∼50% immunoreactive cells and 3 for 50% or more immunoreactive cells. Additionally, the staining intensity was scored as 0 (negative), 1 (weak), 2 (moderate), or 3 (strong). The final immunostaining score was defined as the sum of both scores, which was categorized as negative (0), weak (1 or 2), moderate (3 or 4), and strong (5 or 6).

### Plasmids and reagents

Short hairpin RNA (shRNA) and small interfering RNA (siRNA) were synthesized by Sangon Biotech Company (Shanghai, China). shRNA and siRNA targeting sequences were listed in Tables S5 and S6. siRNA was transfected into cells using GTR Reagent (D16326010, Golden Trans Technology, China) according to the manufacturer’s instructions. Full-length cDNAs of genes were cloned into PCMV and PCDH vectors using a standard molecular cloning protocol.

### Lentiviral infection

293T/17 cells were seeded in 60 mm culture dishes until the confluence reached 90% for transfection: 250 μl serum-free DMEM medium, 1.44 μg pMD2.G (envelop plasmid), 2 μg pMDL-G/P-RRE (packaging plasmid), 1 μg pRSV-REV (packaging plasmid), 5.12 μg target plasmid and 10 μl Lipo8000 (C0533, Beyotime Biotechnology) were mixed for each dish and left at room temperature for 5 min. The mixture was added to the dishes and incubated for 5 h in an incubator. Next, the medium was replaced with DMEM medium with 10% fetal bovine serum and continued to be cultured. We collected virus-containing supernatants from 293T/17 cells at 36 h, 48 h, and 60 h after transfection. The collection was centrifuged at 2000 rpm for 5 min, and the supernatant was collected for infection. Tumor cells were plated in 6-well plates and grown to 60% confluency for infection: 1 ml 1640 medium with 10% fetal bovine serum, 1 ml viral supernatant, and 20 μg polybrene were added to each well for 12 h before being replaced with fresh medium. 72 h after the lentiviral infection, cells were selected in the presence of puromycin (AGS and HGC-27: 2 μg/ml, MKN-74: 2.5 μg/ml) or hygromycin (AGS: 200 μg/ml, HGC-27: 250 μg/ml) for 1 week.

### Cell counting

AGS, HGC-27 and MKN74 cells were collected at least 48 h after completing antibiotics selection following lentiviral infection. When the cells reached 70% to 90% confluence, they were seeded into 6-well plates at the density of 1.5 × 10^5^ cells/well (AGS), 1 × 10^5^ cells/well (HGC-27), or 1.875 × 10^5^ cells/well (MKN-74). A continuous 6-day cell growth curve was drawn.

### Colony formation assay

Lentivirus-infected AGS, HGC-27, or MKN74 cells were collected, resuspended at a concentration of 1000 cells/ml, and then seeded in a 12-well plate (1 ml/well). The cells were cultured for 10 days to form colonies. At the completion of the culture, cells were washed with PBS, fixed with 1 ml Carnoy’s fixative (methanol: glacial acetic acid = 3: 1), and stained by 500 ml a crystal violet staining solution (C0121, Beyotime Biotechnology). Visible colonies in 12-well plates were recorded by a stereoscopic microscope (Leica).

### Flow cytometry

Cell cycle distributions were determined using a cell cycle detection kit (KGA512, KeyGEN BioTECH). Cells were harvested during the exponential growth phase. The cells were washed with PBS, fixed in 70% ethanol, and stored at −20 °C for 2 h. The fixed cells were washed twice in 500 μl PBS and the cell pellet was then resuspended with 0.5 ml of a mixture containing propidium iodide and RNase and incubated at room temperature for 30 min protected from light. Cell cycle distributions were analyzed using a flow cytometer (BD) and Modfit software.

### EdU immunofluorescence

Lentivirus-infected AGS, HGC-27 or MKN74 cells were seeded in a 96-well plate and were incubated overnight before adding the BeyoClick EdU-488 detection reagent (C0071S, Beyotime Biotechnology). Levels of EdU incorporation were assessed with an inverted fluorescence microscope (Nikon).

### TUNEL assay

Lentivirus-infected AGS and HGC-27 cells were collected, and resuspended before being seeded in a 96-well plate. After overnight culture, cells were rising twice with PBS, followed by fixation with 4% paraformaldehyde for 15 min and permeabilization in 0.25% Triton-X 100 for 20 min. TUNEL assays were carried out according to the manufacturer's instructions (C1098, Beyotime Biotechnology, China).

### Chromatin immunoprecipitation (ChIP)

HGC-27 cells were grown to 90% confluency. Protein-DNA complexes were cross-linked with 1% formaldehyde for 10 min, followed by the addition of 0.125 M glycine for 5 min. Cells were lysed and sonicated. Cell lysates were diluted and incubated with anti-E2F1 antibody (NBP2-67899, Novus, 5 μg), anti-E2F7 antibody (ab245655, Abcam, 5 μg), and normal rabbit IgG antibody (2729, CST, 2 μg) at 4 °C overnight. The mixture of cell lysates and antibodies was incubated with agarose beads (16–157, Millipore) at 4 °C for 5 h. Immunocomplexes bound by the beads were washed and eluted using an elution buffer. The input and eluted samples were heated at 65 °C overnight to reverse the formaldehyde cross-links. DNA was purified and used for qPCR using primers designed to span the specific binding sites. The ChIP-qPCR primers for MYBL2, E2F1, and γ-tubulin promoters were listed in Table S7.

### Dual luciferase reporter assay

The MYB-PGL3 reporter vector was constructed by Sangon Biotech Company by inserting a synthesized MYBL2 promoter region from −670 bp to −170 bp into a PGL3-basic vector. AGS and HGC-27 cells were co-transfected with MYB-PGL3 plasmid, E2F1 or E2F7 overexpression plasmid, and TK plasmid. Renilla and firefly luciferase report activities were assayed using the dual luciferase kit (E1910, Promega).

### Tet-On gene expression system

A Tet-On system was used to generate several AGS sublines permitting inducible overexpression of E2F1 or inducible knockdown of E2F1 in the presence of doxycycline. The cells were cultured in 10% FBS medium containing doxycycline (D9891, Sigma-Aldrich, USA, 2 μg/ul) for at least 5 days, and needed to be replaced with a fresh medium containing doxycycline every 2 days.

### Hematoxylin and eosin (H&E) staining

Tumor tissues were fixed with 4% paraformaldehyde and embedded in paraffin. Paraffin-embedded sections were placed at 60 °C for 3 h, and then placed into the dimethylbenzene for 15 min. Subsequently, the sections were rehydrated by placing them into 100%, 95%, 90%, 80%, and 70% alcohol solutions in sequence for 5 min each. Rehydrated sections were stained with hematoxylin for 5 min, and washed with ddH_2_O for 5 min. After washing with 3% hydrochloric acid in 95% ethyl alcohol and ddH_2_O for 30 s, respectively, the sections were stained with eosin for 5 min. Finally, the sections were dehydrated, mounted, and observed under an inverted microscope (Nikon).

### Tumor xenograft assay

Cell suspensions (5 × 10^6^ cells) of AGS cells of various groups in a total volume of 100 μl PBS were injected subcutaneously into the left and right flanks of 5-week-old female BALB/C nude mice (Si Pei Fu) (five mice per group, randomly grouping design). The tumor onset of every mouse was recorded. The tumor volumes were measured using a slide caliper and recorded every 3 days from the tumor onset of each mouse. Tumor volume was calculated with the following formula: volume = 0.5 × tumor length × tumor width^2^. At the end of the experiment (48 or 42 days after inoculation), tumors were collected, weighed, and photographed. Each group received doxycycline in the drinking water (2 mg/ml) from the first day after injection of AGS Tet-On cells. The bottles of water were changed every 3 days. All mice were housed in the SPF animal facility of China Medical University in a pathogen-free environment with controlled temperature and humidity. The work was approved by The Institutional Animal Care and Use Committee and the Laboratory Animal Ethics Committee of China Medical University (IACUC-2023278).

### Cell immunofluorescence

Cells were fixed in 4% paraformaldehyde, blocked in 5% bovine serum albumin, and permeabilized in 0.1% Triton X-100 solution at room temperature. Then cells were incubated with primary antibodies against E2F7 (24489-1-AP, Proteintech, 1: 150) at 4 °C overnight, washed with PBS before being incubated at room temperature for 2 h with a Cy3 conjugated goat anti-rabbit secondary antibody (BA1032, BOSTER). Cells were counter-stained with DAPI and imaged under a fluorescence microscope.

### Statistical analysis

All statistical analyses were performed using GraphPad Prism nine software. Quantitative data comparison between two groups was analyzed by independent sample *t* test (or estimation statistics) or paired *t* test, following confirmation of normal distribution with the Shapiro-Wilk test. The differences in the growth curves between two groups were assessed using two-way ANOVA with repeated measures. Pearson correlation coefficient analysis was used to analyze the correlation between gene expression. Kaplan Meier plotter was used for survival analysis. The difference between two groups in the percentage of nucleocytoplasmic distribution was analyzed by chi-square test. Differences between groups were considered significant at *p* < 0.05. The results were presented as mean ± standard deviation (SD) from at least three independent biological experiments.

## Data availability

All data that support the findings in this study are available from the corresponding author upon reasonable request.

## Supporting information

This article contains supporting information (26, 62, 63, 64).

## Conflict of interest statement

The authors declare that they have no conflicts of interest with the contents of this article.

## Figures and Tables

**Supplementary Figure S7:**