# Supplementary material for: E2F1 and E2F7 regulate gastric cancer cell proliferation, respectively, through transcriptional activation and transcriptional repression of MYBL2
Source: J Biol Chem. 2024 Nov 27;301(1):108027. doi: 10.1016/j.jbc.2024.108027 (PMC11731210; doi:10.1016/j.jbc.2024.108027)
Supplement: Supplementary Figure legends and Tables [file mmc1.pdf]

E2F1 and E2F7 regulate gastric cancer cell proliferation respectively through transcriptional activation and transcriptional repression of MYBL2

Tianyi Wu[1], Fengli Jiang[1, 2], Fan Wu[1, †], Guoliang Zheng[1, 3], Yang Li[1, 2], Lizhao Wu[1] *

List of the material:

1. **Supplementary figure legends**
2. **Supplementary tables**

Supplementary figure legends

Figure S1. Quantitative analysis of WB results (n = 3) in Fig. 2A (A), Fig. 2B (B), Fig. 2G (C), Fig. 3G (D), Fig. 3H (E). *F,* Quantitative analysis of WB results in Fig. 5A (n = 3, (**) $P < 0.01$ / (*) $P < 0.05$ vs. tet OE-E2F1 + tet shNC group, #### $P < 0.0001$ / # $P < 0.05$ vs. tet OE-E2F1 + tet shMYBL2 group). *G,* Quantitative analysis of WB results in Fig. 5F (n = 3, (*) $P < 0.05$ vs. shE2F7 + tet shNC group, #ns not significant vs. shE2F7 + tet shMYBL2 group).

Figure S2. Changes in cell growth curves drawn by cell counting after E2F1, E2F7 or MYBL2 knockdown by targeting sequences with lower efficiencies in AGS and HGC-27 cells (n = 4).

Figure S3. Effects of E2F1 overexpression on GC cell proliferation and apoptosis. *A,* Protein levels of E2F1, Ki67 and PCNA in AGS cells were evaluated by WB (n = 3). *B,* Cell growth curves drawn by cell counting after *E2F1* overexpression in AGS cells (n = 4). *C,* Effect of E2F1 overexpression on the colony forming ability of AGS cells were assessed by a colony formation assay (n = 3). *D,* Cell cycle distributions after E2F1 overexpression in AGS cells were evaluated by flow cytometry (n = 3). *E,* Proliferation changes of AGS cells after E2F1 overexpression were assessed by EdU immunofluorescence staining (n = 3). Scale bar: 100 µm. *F,* Apoptosis of AGS cells after E2F1 overexpression was assessed by a TUNEL assay (n = 3). Scale bar: 100 µm.

Figure S4. Effects of MYBL2 knockdown upon E2F1 overexpression on apoptosis of AGS cells were assessed by a TUNEL assay (n = 3, scale bar: 100 µm, (***) $P < 0.001$ vs. tet OE-E2F1 + tet shNC group).

Figure S5. Overexpression of MYBL2 attenuates the tumor-suppressing effects of E2F1 knockdown in GC cells. *A,* E2F1 and MYBL2 protein levels in shNC + Vector, shE2F1 + Vector, shE2F1 + OE-MYBL2, and shNC + OE-MYBL2 groups of AGS cells were evaluated by WB (n = 3). *B,* Cell growth curves were plotted using a cell counting assay in AGS cells (n = 4). *C,* Changes in colony forming ability were evaluated by a colony formation assay in AGS cells (n = 3, (***) $P < 0.001$ vs. shE2F1 + Vector group, # $P < 0.05$ vs. shE2F1 + OE-MYBL2 group). *D,* Cell cycle distributions were assessed by flow cytometry in AGS cells (n = 3, (**) $P < 0.01$ vs. shE2F1 + Vector group). *E,* Cell proliferation levels were assessed by EdU immunofluorescence assay in AGS cells

(n = 3, (***) *P* < 0.001 vs. shE2F1 + Vector group). Scale bar: 100 μm. *F,* E2F1 and MYBL2 protein levels in shNC + Vector, shE2F1 + Vector, shE2F1 + OE-MYBL2, and shNC + OE-MYBL2 groups of HGC-27 cells were evaluated by WB (n = 3). *G,* Cell growth curves were plotted using a cell counting assay in HGC-27 cells (n = 4). *H,* Colony forming abilities were evaluated by a colony formation assay in HGC-27 cells (n = 3, (****) *P* < 0.0001 vs. shE2F1 + Vector group, ## *P* < 0.01 vs. shE2F1 + OE-MYBL2 group). *I,* Cell cycle distributions were assessed by flow cytometry in HGC-27 cells (n = 3, (**) *P* < 0.01 vs. shE2F1 + Vector group). *J,* Cell proliferation levels were assessed by EdU immunofluorescence assay in HGC-27 cells (n = 3, (**) *P* < 0.01 vs. shE2F1 + Vector group). Scale bar: 100 μm.

Figure S6. Overexpression of MYBL2 attenuates the tumor-suppressing effects of E2F7 overexpression in GC cells. *A,* E2F7 and MYBL2 protein levels in Vector 1 + Vector 2, OE-E2F7 + Vector 2, OE-E2F7 + OE-MYBL2, and Vector 1 + OE-MYBL2 groups of AGS cells were evaluated by WB (n = 3). *B,* Cell growth curves were plotted using a cell counting assay in AGS cells (n = 4). *C,* Changes in colony forming ability were evaluated by a colony formation assay in AGS cells (n = 3, (**) *P* < 0.01 vs. OE-E2F7 + Vector 2 group, # *P* < 0.05 vs. OE-E2F7 + OE-MYBL2 group). *D,* Cell cycle distributions were assessed by flow cytometry in AGS cells (n = 3, (**) *P* < 0.01 / (***) *P* < 0.001 / (****) *P* < 0.0001 vs. OE-E2F7 + Vector 2 group). *E,* Cell proliferation levels were assessed by EdU immunofluorescence assay in AGS cells (n = 3, (*) *P* < 0.05 vs. OE-E2F7 + Vector 2group). Scale bar: 100 μm. *F,* E2F7 and MYBL2 protein levels in Vector 1 + Vector 2, OE-E2F7 + Vector 2, OE-E2F7 + OE-MYBL2, and Vector 1 + OE-MYBL2 groups were evaluated by WB in HGC-27 cells (n = 3). *G,* Cell growth curves were plotted using a cell counting assay in HGC-27 cells (n = 4). *H,* Colony forming abilities were evaluated by a colony formation assay in HGC-27 cells (n = 3, (***) *P* < 0.001 vs. OE-E2F7 + Vector 2 group, # *P* < 0.05 vs. OE-E2F7 + OE-MYBL2 group). *I,* Cell cycle distributions were assessed by flow cytometry in HGC-27 cells (n = 3, (**) *P* < 0.01 / (***) *P* < 0.001 vs. OE-E2F7 + Vector 2 group). *J,* Cell proliferation levels were assessed by EdU immunofluorescence assay in HGC-27 cells (n = 3, (****) *P* < 0.0001 vs. OE-E2F7 + Vector 2 group). Scale bar: 100 μm.

Figure S7. Estimation plots illustrating the nucleocytoplasmic distribution of E2F7 in relation to the mRNA levels of target genes and the protein levels of Ki67. *A,* The mRNA levels of E2F1 in 30 GC samples with different nucleocytoplasmic distributions of E2F7 protein. *B,* The mRNA levels of MYBL2 in 30 GC samples with different nucleocytoplasmic distributions of E2F7 protein. *C,* IHC staining scores of Ki67 in 30 GC samples with different nucleocytoplasmic distributions of E2F7 protein. Left panels of (A-C): Comparison between the group with nuclear and cytoplasmic distribution of E2F7 and the group with primarily nuclear distribution of E2F7 (N/C - Nu). Right panels: Comparison between the group with primarily cytoplasmic distribution of E2F7and the group with primarily nuclear distribution of E2F7 (Cyto - Nu).

Supplementary tables

Table S1. E2F1 immunohistochemical scores

Group	No. of cases	E2F1 protein levels				*P* value
		Negative (0)	Weak (1&2)	Moderate (3&4)	Strong (5&6)	
Adjacent tissues	30	1	21	5	3	< 0.001
GC	30	0	0	7	23	

Table S2. E2F7 immunohistochemical scores

Group	No. of cases	E2F7 protein levels				*P* value
		Negative (0)	Weak (1&2)	Moderate (3&4)	Strong (5&6)	
Adjacent tissues	30	6	22	2	0	< 0.001
GC	30	0	2	7	21	

Table S3. MYBL2 immunohistochemical scores

Group	No. of cases	MYBL2 protein levels				*P* value
		Negative (0)	Weak (1&2)	Moderate (3&4)	Strong (5&6)	
Adjacent tissues	30	4	24	1	1	< 0.001
GC	30	0	2	14	14	

Table S4. RT-qPCR primers

Primer	Sequence (5'~3')	Reference
E2F1 Forward	ATGAGACCTCACTGAATCTGACCACC	-
E2F1 Reverse	AGGTCCTGGGTCAACCCCTC	
E2F7 Forward	GAAGCGCCTCTGTGAGGAGA	-
E2F7 Reverse	CTTCTGCAGCAGGGAGTTGG	
MYBL2 Forward	CCGGAGCAGAGGGATAGCA	（62）
MYBL2 Reverse	CAGTGCGGTTAGGGAAGTGG	
GAPDH Forward	ACAACTTTGGTATCGTGGAAGG	-
GAPDH Reverse	GCCATCACGCCACAGTTTC	

Table S5. shRNA targets

Target	Sequence (5’~3’)	Reference
shE2F1-1	CAGGATGGATATGAGATGGGA	（63）
shE2F1-2	CCTGAGGAGTTCATCAGCCTT	（63）
shE2F7-1	CGCCTCTATGACATAGCCAAT	（64）
shE2F7-2	GCAACAGCAAACTCTCTTGTT	-
shMYBL2-1	CCCAGATCAGAAGTACTCCAT	-
shMYBL2-2	GCTTGGTGTGACCTGAGTAAA	-

Table S6. siRNA sequences

siRNA	Sequence (5'~3')
siNT sense	UUCUCCGAACGUGUCACGUTT
siNT antisense	ACGUGACACGUUCGGAGAATT
siE2F1 sense	CAUCCAGCUCAUUGCCAAGAATT
siE2F1 antisense	UUCUUGGCAAUGAGCUGGAUGTT
siE2F7 sense	CAGAAGAGCGAGGUCGUAAUU
siE2F7 antisense	UUACGACCUCGCUCUUCUGUU

Table S7. ChIP-qPCR primers

Primer	Sequence (5’~3’)	Reference
MYBL2 promoter-F	CTGGAGGGTCTGGGCAGGTG	-
MYBL2 promoter-R	CGCGGGTTGAAGCACTTTTC	
E2F1 promoter-F	CTGCCTGCAAAGTCCCGGCCACTT	（26）
E2F1 promoter-R	AGGAACCGCCGCCGTTGTTCCCGT	
γ-Tubulin promoter-F	ATGGAGGGATGAATGGTTATGC	（26）
γ-Tubulin promoter-R	CTTTTTGGGTCTGGCTTCTTTCAC	
